# Impact of ketogenic diets on cancer patient outcomes: a systematic review and meta-analysis

**DOI:** 10.3389/fnut.2025.1535921

**Published:** 2025-07-18

**Authors:** Meiying Zhang, Qing Zhang, Sheng Huang, Yifu Lu, Mengyun Peng

**Affiliations:** ^1^Department of Diagnostic Imaging, National Cancer Center/National Clinical Research Center for Cancer/Cancer Hospital, Chinese Academy of Medical Sciences and Peking Union Medical College, Beijing, China; ^2^Department of Thoracic Surgery, National Cancer Center/National Clinical Research Center for Cancer/Cancer Hospital, Chinese Academy of Medical Sciences and Peking Union Medical College, Beijing, China; ^3^School of Nursing, Suzhou Medical College, Soochow University, Suzhou, China; ^4^Medical College, Shantou University, Shantou, China

**Keywords:** tumor, ketogenic diet, diets, ketogenic, systematic review and meta-analysis

## Abstract

**Background:**

The ketogenic diet, characterized by high fat, moderate protein, and extremely low carbohydrate intake, has been widely used as a medical treatment for various conditions and has gained increasing attention in recent years due to its health benefits.

**Objectives:**

This study aims to investigate the effectiveness of a ketogenic diet on outcomes in cancer patients compared to conventional non-ketogenic diets.

**Materials and methods:**

Studies that assigned cancer patients to either a ketogenic diet or a standard diet control group were included. Two reviewers independently extracted and analyzed the data.

**Results:**

This meta-analysis revealed that the ketogenic diet significantly reduced fat mass, visceral fat, insulin levels, blood glucose, fatigue, and insomnia compared to a non-ketogenic diet while improving low-density lipoprotein (LDL) cholesterol, total cholesterol, thyroid-stimulating hormone (TSH) levels, protein uptake, ketosis events, emotional function, and social function. Furthermore, the ketogenic diet induced ketosis by increasing β-hydroxybutyrate levels.

**Conclusion:**

The ketogenic diet was found to improve cancer patients’ outcomes more effectively than non-ketogenic diets. Notably, C-reactive protein levels showed greater improvement when the intervention lasted more than 12 weeks, with a diet composition of 2–4% carbohydrates, 16–18% protein, and 80–85% fat.

**Systematic review registration:**

(https://www.crd.york.ac.uk/PROSPERO/view/CRD42024553878) PROSPERO CRD4202455387.

## Introduction

The World Health Organization (WHO) defines cancer as a group of diseases characterized by the uncontrolled growth of abnormal cells. According to global cancer data (1), there were 19.98 million new cancer cases in 2022, with a significant increase in less developed countries (2). Lung, breast, and colorectal cancers were the most common types (3). Cancer places a substantial burden on healthcare systems and is the second leading cause of death worldwide, making it one of the most pressing public health challenges (4).

Chemotherapy, radiotherapy, and surgery are the mainstays of conventional cancer treatments, but they often cause significant side effects (5). Recently, increasing attention has been directed toward the ketogenic diet (KD), a high-fat, low-carbohydrate diet, for its potential role in managing various types of cancer (6–9). Studies have suggested that KD may inhibit tumor growth by altering cellular metabolism and improve the tolerance of normal cells to radiotherapy and chemotherapy (10). Furthermore, KD may enhance the effectiveness of PD-1 blockade, a type of immunotherapy (11).

Several controlled clinical trials (CCTs) have investigated the effectiveness of KD in cancer treatment, with a focus on cancers such as glioma and breast cancer (12–18). These trials have demonstrated that KD may inhibit tumor progression by modulating metabolic pathways and enhancing the efficacy of standard treatments, such as chemotherapy and radiotherapy. Additionally, KD has been reported to improve patient tolerance to these therapies, reducing side effects. Based on these findings, researchers suggest that KD could be a safe and effective complementary therapy in cancer treatment, providing a potential new avenue for improving patient care. Despite ongoing debates about its overall effectiveness, the ketogenic diet continues to be explored as a potential adjuvant therapy in oncology.

The traditional KD, characterized by a 4:1 ketogenic ratio, is composed of approximately 90% fat, 8% protein, and 2% carbohydrates. However, several studies have suggested that a low-carbohydrate ketogenic diet (LCKD), with a composition of 70–80% fat, 5–10% carbohydrates, and 10–20% protein, may offer even greater benefits (19). More robust evidence is needed to identify the optimal KD composition for maximizing therapeutic outcomes in cancer treatment.

This meta-analysis aims to address this gap by collecting data from a limited number of studies examining KD as an adjuvant therapy in cancer patients. The analysis focuses on evaluating the significance of KD’s effects on body composition, lipid profile, immunologic factors, internal secretion, liver and kidney function, dietary intake, quality of life, and other factors such as ketosis events and adverse reactions. Additionally, we aim to explore how patients’ age, intervention duration, and dietary intervention ratios impact KD’s anti-tumor effects through subgroup analyses, providing further insights into its therapeutic potential.

## Materials and methods

This systematic review was registered with PROSPERO (CRD 42024553878) and conducted in accordance with the Preferred Reporting Items for Systematic Reviews and Meta-Analyses (PRISMA) guidelines (20).

### Search strategy and inclusion criteria

Medical Subject Headings (MeSH) and text words related to KD and cancer were used to identify the included studies. The search strategy was executed across six databases (PubMed, Web of Science, EMBASE, CINAHL, Medline, and the Cochrane Library) from inception through June 2024 (Supplementary Table). To ensure comprehensive coverage, a manual search of references was also conducted to identify potentially eligible studies. Screening and study selection were conducted independently by two authors (M Zhang and M Peng).

Studies were selected based on the PICOS principles (Table 1). Two authors (M. Zhang and M. Peng) independently reviewed the titles and abstracts of the selected articles, without being blinded to the authors or article titles. Full-text articles deemed potentially eligible were subsequently retrieved for further assessment. Any disagreements that arose during the selection process were resolved through consensus (Q. Zhang), with decisions made based on predefined inclusion and exclusion criteria.

**Table 1:** Inclusion and exclusion criteria.

Study participants (P)	Patients diagnosed with any kind of cancer or tumor.
Intervention (I)	Studies that used KD as an intervention.
Control (C)	A regular or standard diet in the control group.
Outcomes (O)	Body composition (fat mass, visceral fat mass, skeletal muscle mass, body cell mass), lipid profile (HDL cholesterol, LDL cholesterol, triglycerides, total cholesterol), immunologic factors (insulin, blood glucose, C-reactive protein, IGF-1, TNF-α, IL-10,serum β-hydroxybutyrate), internal secretion (TSH, FT3), liver and kidney function (GGT, creatinine, urea), diet amount(energy uptake, protein uptake, time to exhaustion), quality of life (quality of life score, emotional function, fatigue, insomnia, social function, future perspective, systemic therapy side effects) and other aspects (ketosis event, adverse event).
Study design (S)	Contrary to our original study protocol specification to only consider RCTs, we more generally decided to consider non-RCTs for meta-analysis, because the randomization is not always practical in certain cancer patients who might have their own diet preferences.

### Data extraction and quality assessment

The two authors (M. Zhang and M. Peng) independently extracted data following the PICOS principle. Any discrepancies were resolved through discussion with the third author (Q Zhang), and decisions were made based on predefined inclusion/exclusion criteria and methodological quality considerations. The extracted data included the first author, publication year, study design, age of participants, cancer type, intervention and control diets, number of participants, duration of intervention, and outcome. Numerical data from the figures were extracted using Engauge Digitizer. When outcomes were reported in different formats, the results were standardized (mean ± standard deviation).

The risk of bias was assessed in accordance with the Cochrane Handbook guidelines, with a focus on key areas, including selection bias (21, 22). Discrepancies were resolved through discussion. Additionally, the quality of evidence was rated as high, moderate, low, or very low using the GRADE approach, which evaluates studies based on risk of bias, consistency, directness, precision, and publication bias (23).

### Statistical analysis

Meta-analyses were conducted using RevMan software. The Cochran–Mantel–Haenszel method was applied for categorical data, and the inverse-variance method was used for continuous outcomes. To ensure consistency, data reported as medians, means, standard deviations (SD), or interquartile ranges (IQR) were converted to mean ± SD using established formulas (24–26). For studies reporting standard errors of the mean (SEM), SDs were calculated using the formula: SD = SEM × √N (27).

Effect sizes were expressed as standardized mean differences (SMDs) with 95% confidence intervals (CIs). Heterogeneity among studies was assessed using the I^2^ statistic. A random-effects model was used if I^2^ > 50%; otherwise, a fixed-effects model was applied. In cases of substantial heterogeneity, sensitivity analyses were performed by examining individual studies. Statistical significance was set at a *p*-value of < 0.05.

## Result

### Study selection

**Figure 1:**
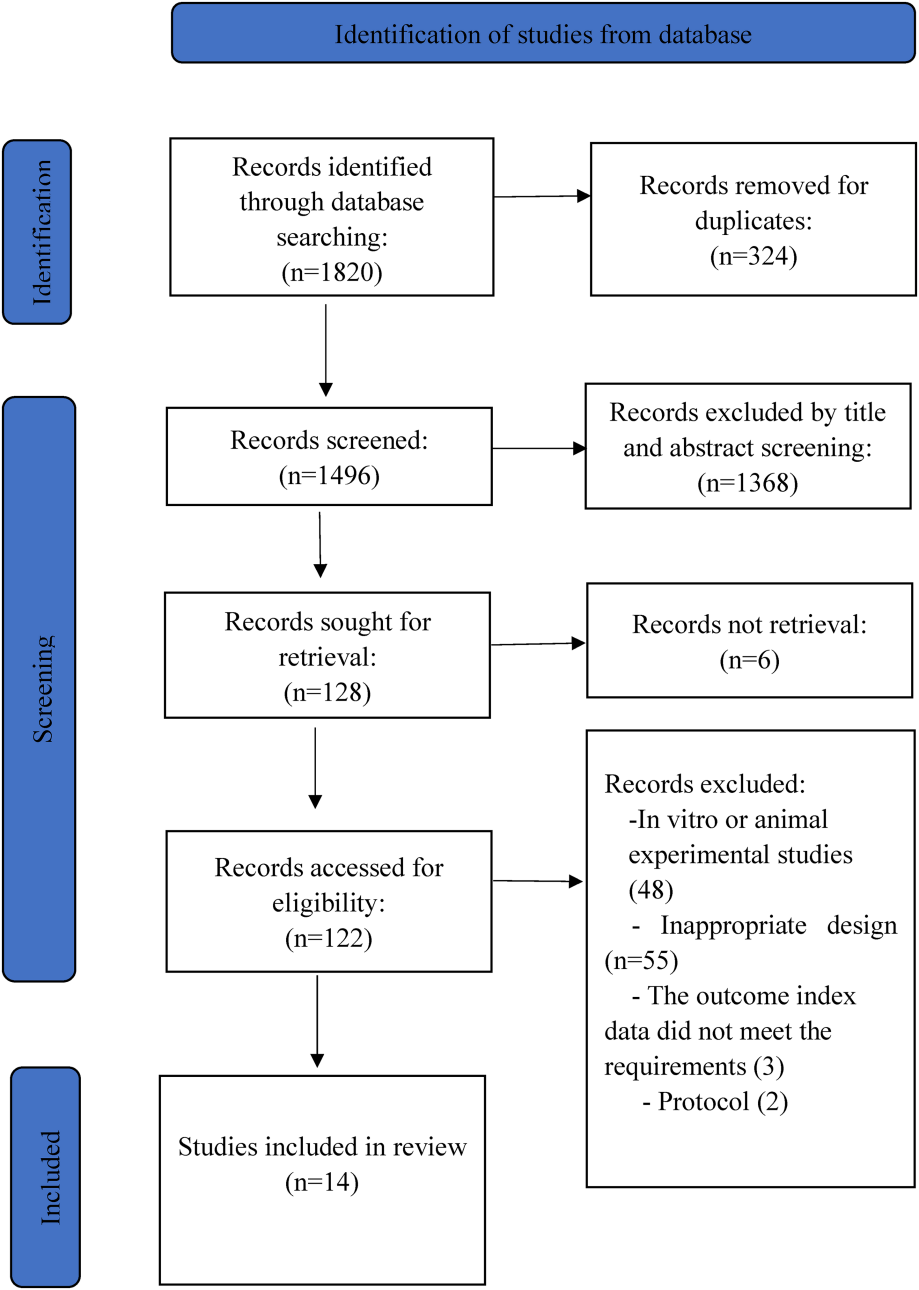
PRISMA flow chart.

The initial database search yielded 1,820 citations. After removing duplicates, 1,496 articles remained for title and abstract screening. Of these, 122 full-text articles were selected for further evaluation. Ultimately, 108 articles were excluded: 48 were *in vitro* or animal studies, 55 had inappropriate designs, and five studies were excluded due to outcomes data not meeting requirements (*n* = 3) or being protocols (*n* = 2). A total of 14 clinical trials and 16 publications were included (12, 13, 15, 16, 18, 28–36). Figure 1 describes the literature search and study selection process.

### Study characteristics

The included studies were conducted in Germany (*n* = 5) (13, 15, 16, 29, 30), the USA (*n* = 4) (18, 28, 32, 33), Iran (*n* = 3) (12), Korea (*n* = 2) (31, 34), Indonesia (*n* = 1) (35), and Brazil (*n* = 1) (36). The publication dates ranged from 2018 to 2020. A total of 11 trials were RCTs (12, 18, 28–33, 35), while five trials were non-RCTs (13, 15, 16, 34, 36), with the follow-up periods ranging from 6 days to 24 weeks. In this review, a KD was compared with either a standard or normal diet (12, 13, 15, 16, 29–31, 36) or other dietary approaches, including a low-carb diet (13), a general hospital diet (18, 33–35), or the American Cancer Society diet (28, 32).

In one study (13), the control group consisted of two separate groups: a standard group and a low-carbohydrate diet group. Participants across the included studies had various types of cancer, such as breast cancer (12, 13, 15), glioma (29, 30, 36), ovarian/endometrial cancer (29, 30), pancreatic biliary tract cancer (31, 34), carcinoma of the rectum (16), colorectal cancer (35). General characteristics are presented in Table 2.

**Table 2:** Study characteristics and effect of ketogenic diet on outcomes of cancer patients.

General information												Data					
Reference	Country	Design	Type of cancer	Concurrent Treatment	Age (mean ± sd) years (I/C)	Sex, Participants Number of I/C		Intervening Measure		Duration(Week or day)	Outcome	Intervention			Control		
						T	C	T	C			Mean	SD	Number	Mean	SD	Number
Cohen et al. (28)	USA	RCT	Ovarian or Endometrial cancer	Chemotherapy	61.5 ± 8.5/58.6 ± 11.7	F 25	F 20	KD:5% carbohydrate (≤20 g/d),25% protein (≤100 g/d),70%fat (≥125 g/d)	ACS: high-fiber, low-fat	12 weeks	Fat mass	32.7	14.9	25	41.2	19.6	20
											Visceral fat mass	975	754.5	25	1,024	785.31	20
											Insulin	6.7	4.1	23	12.1	6.7	20
											β-hydroxybutyrate	0.91	0.73	23	0.25	0.18	20
											Blood glucose	93	15.83	23	98.75	11.8	20
											CRP	2	1.44	23	3	1.34	20
											IGF-1	100.7	44	23	111	59.9	20
Voss et al. (29, 30)	Germany	RCT	Glioblastoma or Gliosarcoma	Chemotherapy	57.74 ± 8.15/55.50 ± 11.45	M/F 20	M/F 20	KD-IF: Carbohydrate limit is 50 g/day. The patient fasted on day 4 with 6 unlimited fluid intake. From day 10, patients no longer have dietary restrictions.	SD: 30 kcal/kg (about 60–80 g fat, 5 g /kg carbohydrate and 0.8 g /kg protein)	6 days	Insulin	6.9	3.7	22	17.25	19.17	18
											IGF-1	179.9	79.7	22	219.6	104.3	15
											Urea	30.67	8.4	20	30.36	9.67	21
Kang et al. (31)	Korea	RCT	Pancreatic biliary carcinoma	Operation	58.3 ± 7.6/66.3 ± 9.8	M 5\u00B0F 4	M 6\u00B0F 3	LCKD:80% kcal from fat, ketogenic ratio of 1.75:1	GD	4 weeks	TNF-α	498.3	813.3	9	666.52	829.14	9
											Creatinine	232	93.5	9	150.2	49.3	9
											β-hydroxybutyrate	0.29	0.17	9	0.27	0.15	9
											Ketone body	1.67	0.952	9	2.3	1.59	9
											Insulin	11.58	10.52	9	6.14	2.63	9
											Blood glucose	132.14	246.43	9	106.25	77.68	9
Cohen et al. (32)	USA	RCT	Ovarian or Endometrial cancer	Chemotherapy	61.5 ± 8.5/58.6 ± 11.7	F 25	F 20	KD:70% fat, 25% protein, 5% carbohydrate	ACS: high-fiber, low-fat	12 weeks	Total Cholesterol	214	60	23	208	44	20
											HDL-Cholesterol	72	13	23	65	15	20
											LDL-Cholesterol	123	55	23	119	40	20
											Triglycericle(TG)	97	33	23	111	46	20
											Energy uptake	1,239	304	23	1,533	304	15
											Protein uptake	29	0.7	23	18.5	1	15
Khodabakhshi et al. (46)	Iran	RCT	Breast cancer	Chemotherapy	44.8 ± 8.4/45.2 ± 15.0	M/F 30	M/F 30	KD of medium chain triglyceride (MCT) (containing 6% caloric content from CHO, 19% protein, 20% MCT, 55% fat)	SD	12 weeks	Fat mass	29.1	7.1	30	30.8	7.5	30
											Blood glucose	84.5	11.3	30	105.2	15.8	30
											Insulin	5.7	4	30	6.9	4.5	30
											IGF-1	133	61	30	150	48	30
											Ketone body	0.923	0.699	30	0.007	0.026	30
											Creatinine	0.77	0.09	30	0.86	0.15	30
											TNF-α	18	8.6	30	17.3	7.3	30
Augustus et al. (33)	Trinidad	RCT	Breast cancer/Prostate/Colon/Rectum/Lung cancer/Cervix	Chemotherapy	49.80 ± 6.72/51.80 ± 4.18	M/F 17	M/F 20	KD = approximately 10% CHO, 15% Proteins, and 75% Fats	Usual diet	16 weeks	Blood glucose	74.8	6.204	17	84.4	7.358	20
											QoL score	36.85	7.58	17	60.45	14.9	20
Khodabakhshi et al. (12, 47)	Iran	RCT	Breast cancer	Chemotherapy	44.8 ± 8.4/45.2 ± 15.0	M/F 30	M/F 30	KD of medium chain triglyceride (MCT) (containing 6% caloric content from CHO, 19% protein, 20% MCT, 55% fat)	SD:55% CHO, 15% protein and 30% fat	12 weeks	Tumor size	27	25	30	34	26	30
											Energy uptake	154.32	141.89	30	1603.8	65.68	30
											Protein uptake	57.12	5.15	30	69.95	6.37	30
											CRP	12	13	30	14.3	14	30
											IL-10	11.1	4.7	30	10.1	4.3	30
										6 weeks	TNF-α	19	9.1	30	16.4	6	30
											Insulin	6.1	6.7	30	9.1	9.6	30
											CRP	11	13	30	18.6	18	30
											IL-10	10.6	4.5	30	10	4.5	30
											QoL score	75	20	30	62	20	30
											Emotional function	62	23	30	60	21	30
											Social function	91	17	30	87	17	30
											Fatigue	33.95	8.82	30	35.62	5.39	30
											Insomnia	4.88	8.88	30	31.1	12.25	30
											Future perspective	62.08	8.09	30	40.97	16.42	30
											Systemic therapy side effects	40.57	7.84	30	42.12	4.66	30
											IGF-1	140	63	30	136	34	30
Ok et al. (34)	Korea	NRCT	Pancreatobiliary cancer after pancreatectomy	Pancreatectomy	57.8 ± 7.3/66.3 ± 9.8	M/F 10	M/F 9	KD: Carbohydrate: Protein: Fat = 3 ~ 6: 15 ~ 25:70 ~ 80 ketogenic ratio of 1.05 ~ 1.75:1	Carbohydrate: Protein: Fat = 55–65:7–20:15–30	10 days	Fat mass	14.2	6.4	8	17.1	4.9	9
											Triglycericle (TG)	125.7	49.8	10	110.5	51.1	9
											HDL	38.4	8.8	10	42.4	6.9	9
											LDL	108.4	28.3	10	97.4	41.9	9
											Total cholesterol (TC)	173.8	32.1	10	156	48.4	9
											CRP	25.6	36.6	10	10.0	11.8	9
											LOS	12	5.8	10	15.8	11.2	9
											Average meal compliance	69.1	19.6	10	33.9	16.6	9
											Energy uptake	61.31	19	10	38.5	21.9	9
											Dietary overall satisfaction	6.2	1.8	10	3.8	1.1	9
											Urine acetone bodies	50.8	35.1	10	22.2	23.7	9
Kämmerer et al. (13)	Germany	NRCT	Breast cancer	Neoadjuvant chemotherapy	52.5 ± 6.4/51.3 ± 8.1	F 29	F 92	KD: Carbohydrate: Protein: Fat = 2–4%:16–18%:80–85% etogenic ratio of 1.6:1–2:1	LCD: Carbohydrate: Protein: Fat = 20–30%:20–30%: 40–50%	20 weeks	Fat mass	19.5	6.91	19	24.31	7.00	70
											Visceral fat mass	8.5	4.36	19	12.15	5.78	70
											Skeletal muscle mass	40.86	3.68	19	41.97	5.38	70
											BCM: body cell mass	22.02	2.19	19	22.57	3.21	70
											HDL	75.83	22.21	19	67.64	13.71	70
											LDL	150.59	37.38	19	142.13	33.33	70
											CRP	2.05	2.74	19	1.60	2.17	70
											Insulin	12.45	6.85	19	14.7	8.43	70
											IGF-1	13.99	12.27	19	6.94	3.54	70
											TSH	1.83	1.3	19	1.61	0.94	70
											Quality of life score	70.05	22.29	20	65.57	17.39	75
											Emotional function	70.52	26.76	20	64.43	20.88	75
											Fatigue	21.01	23.79	20	35.57	20.88	75
											Insomnia	38.26	26.76	20	64.43	20.88	75
											Time to exhaution	8.55	1.94	19	7.66	1.65	70
											Energy uptake	32.5	1.5	19	24.30	0.7	70
											Protein uptake	1.33	0.07	19	1.20	0.03	70
					52.5 ± 6.4/51.3 ± 8.1	F 29	F 31	KD: Carbohydrate: Protein: Fat = 2–4%:16–18%:80–85% etogenic ratio of 1.6:1–2:1	SD: Carbohydrate: Protein: Fat = 52–62%:16–17%:28–31%	20 weeks	Fat mass	19.5	6.91	19	23.84	8.14	23
											Visceral fat mass	8.5	4.36	19	12.18	4.85	23
											Skeletal muscle mass	40.86	3.68	19	39.71	6.07	23
											BCM: body cell mass	22.02	2.19	19	22.14	3.86	23
											HDL	75.83	22.21	19	65.38	12.96	23
											LDL	150.59	37.28	19	124.48	22.29	23
											CRP	2.05	2.74	19	1.96	1.94	23
											Insulin	12.45	6.85	19	15.82	9.25	23
											IGF-1	13.99	12.27	19	7.83	5.08	23
											TSH	1.83	1.3	19	1.20	0.88	23
											Quality of life score	70.05	22.29	20	64.45	17.13	24
											Emotional function	70.52	26.76	20	56.06	25.68	24
											Fatigue	21.01	23.79	20	48.5	22.83	24
											Insomnia	38.26	26.76	20	37.8	25.68	24
											Time to exhaution	8.55	1.94	19	7.50	1.68	23
											Energy uptake	32.5	1.5	19	26.4	1.2	23
											Protein uptake	1.33	0.07	19	0.98	0.04	23
Klement et al. (15)	Germany	NRCT	Breast cancer	Radiotherapy	52.75 ± 14.14/49.73 ± 12.41	F 29	M 30	KD = 75–80% calories from fat, and limiting carbohydrates to 50 g per day and 10 g per meal	Standard recommendations according to the German Nutrition Society	12 weeks	GGT	17.55	8.14	29	20.31	9.07	30
											Creatinine	0.76	0.11	29	0.84	0.12	30
											Urea	4.74	0.1	29	4.74	0.99	30
											Total cholesterol (TC)	220.27	47.62	29	196.92	33.08	30
											HDL	73.06	24.18	29	67.48	15.19	30
											LDL	141.26	47.38	29	113.82	26.83	30
											Triglycericle (TG)	80.24	24.43	29	108.53	55.63	30
											CRP	3.98	5.95	29	3.34	5.27	30
											IGF-1	187.24	92.53	29	186.46	63.72	30
											Insulin	9.29	7.6	29	8.35	4.97	30
											FT3	2.82	0.36	29	3.05	0.37	30
											TSH	1.76	0.97	29	1.60	0.74	30
											Blood glucose	103.15	16.29	29	96.76	9.31	30
											β-hydroxybutyrate	0.67	0.55	29	0.36	0.63	30
											Quality of life score	83.11	28.39	29	77.1	12.03	30
											Emotional functioning	75	12.34	29	66.69	16.35	30
											Social functioning	79.27	16.45	29	66.69	16.35	30
											Fatigue	34.66	19.19	29	44.18	13.63	30
											Insomnia	37.25	24.68	29	37.27	24.51	30
											Future perspective	62.65	24.68	29	62.73	24.51	30
											Systemic therapy side effects	19.01	9.4	29	24.97	15.17	30
Klement et al. (16)	Germany	NRCT	Rectal cancer	Radiotherapy or Chemotherapy	56.75 ± 10.7/62.25 ± 8.55	M/F 18	M/F 23	KD = 75–80% calories from fat, and limiting carbohydrates to 50 g per day and 10 g per meal	Standard recommendations according to the German Nutrition Society	12 weeks	Blood glucose	108.73	22.51	29	131.27	61.96	23
											Insulin	0.89	5.52	18	20.45	12.5	23
											IGF-1	135.77	47.21	18	175.72	79.33	23
											Total cholesterol(TC)	184.26	28.54	18	182.89	40.7	23
											HDL	64.69	14.27	18	62.79	17.89	23
											LDL	109.47	26.62	18	103.03	35	23
											Triglycericle(TG)	81.32	58.19	18	136.9	36.55	23
											β-hydroxybutyrate	0.86	0.61	18	0.14	0.12	23
											Creatinine	0.87	0.17	18	0.91	0.19	23
											GGT	35.15	38.42	18	49.41	52.63	23
											Urea	5.14	0.88	18	5.93	1.93	23
											CRP	4.33	2.74	18	12.22	18.41	23
											FT3	2.26	0.43	18	3.00	0.47	23
											TSH	6.21	9.17	18	2.79	3.00	32
											QoL score	75.7	11.61	18	64.28	22.04	23
											Emotional function	74.2	18.57	18	64.28	22.04	23
											Social function	83.32	9.27	18	53.98	26.46	23
											Fatigue	36.9	24.75	18	38.14	26.46	23
											Insomnia	33.32	18.57	18	70.78	17.81	23
Budipramana et al. (35)	Indonesia	RCT	Stage-IV colorectal adenocarcinoma	Operation	40–65 range/17–65 range	M/F 12	M/F 12	Very low carbohydrate diet: Carbohydrate: Protein+Fat = 20%:80%	Uommon patients requirement	3 weeks	Creatinine	0.87	0.19	12	0.72	0.21	12
											CRP	3.82	0.46	12	2.96	0.46	12
Freedland et al. (18)	USA	RCT	Recurrent prostate cancer	Radiotherapy	71.25 ± 1.47/70.76 ± 2.56	M 27	M 18	The LCD = limit carbohydrate intake to ≤20 grams/day	Usual diet	24 weeks	Triglycericle(TG)	125.61	17.78	27	95.64	13.45	18
											HDL	46.5	3	27	49.87	6.31	18
											LDL	100	12.52	27	89.31	8.23	18
											Blood glucose	98.63	3.76	27	106.53	5.21	18
											CRP	2.18	0.58	27	2.41	0.78	18
Santos et al. (36)	Brazil	NRCT	Recurrent glioblastoma	Radiotherapy or Chemotherapy	49.5 ± 8.64/44.5 ± 8.06	M/F 9	M/F 8	KD: Carbohydrate: Protein: Fat = 25%: 50%: 25%	SD	12 weeks	Total cholesterol(TC)	220.7	45.5	9	192.6	48	8
											LDL	122.1	27.9	9	116.5	36	8
											Triglyceride (TG)	154.4	93.3	9	99.1	42	8

RCT:randomized controlled trial; NRCT:non randomized controlled trial; ACS: American Cancer Society diet; KD: ketogenic diet; LCD: Low carb diet; SD: standard diet; GD: general hospital diet; CRP: C-reactive protein; FT3: free triiodothyronine; TSH: Thyroid Stimulating Hormone; TNF-α:tumor necrosis factor; GGT: gamma-glutamyl transpeptidase.

### Assessment of the risk of bias

Eight trials included in the meta-analysis were rated as good quality, while four were considered poor quality and two were rated as fair. Seven trials showed a high risk of bias in random sequence generation, and five had significant bias in participant allocation concealment. Blinding was not possible due to the nature of the intervention, so it was excluded from the overall quality evaluation. Outcome assessment blinding was unclear in three studies. Additionally, three trials addressed incomplete outcome data, one had unclear selective reporting bias, and another one had an unspecified bias (Table 3).

**Table 3:** Study characteristics and effect of ketogenic diet on outcomes of cancer patients.

General information												Data					
Reference	Country	Design	Type of cancer	Concurrent Treatment	Age (mean ± sd) years (I/C)	Sex, Participants Number of I/C		Intervening Measure		Duration (Week or day)	Outcome	Intervention			Control		
						T	C	T	C			Mean	SD	Number	Mean	SD	Number
Cohen et al. (28)	USA	RCT	Ovarian or Endometrial cancer	Chemotherapy	61.5 ± 8.5/58.6 ± 11.7	F 25	F 20	KD:5% carbohydrate (≤20 g/d),25% protein (≤100 g/d),70%fat (≥125 g/d)	ACS: high-fiber, low-fat	12 weeks	Ketosis Event	occurred	not occurred	13/10	occurred	not occurred	2/18
Cohen et al. (32)	USA	RCT	Ovarian or Endometrial Cancer	Chemotherapy	61.5 ± 8.5/58.6 ± 11.7	F 25	F 20	KD:5% carbohydrate (≤20 g/d),25% protein (≤100 g/d),70%fat (≥125 g/d)	ACS: high-fiber, low-fat	12 weeks	Adverse Event	occurred	not occurred	13/2	occurred	not occurred	4/96
Voss et al. (29, 30)	Germany	RCT	Glioblastoma or Gliosarcoma	Radiotherapy		M/F 20	M/F 20	KD-IF: Carbohydrate limit is 50 g/day. The patient fasted on day 4 with 6 unlimited fluid intakes. From day 10, patients no longer have dietary restrictions.	SD: 30 kcal/kg (about 60–80 g fat, 5 g /kg carbohydrate and 0.8 g /kg protein)	18 days	Adverse Event	occurred	not occurred	5/20	occurred	not occurred	6/19
Ok et al. (34)	Korea	NRCT	Pancreatobiliary cancer after pancreatectomy	Pancreatectomy	57.8 ± 7.3/66.3 ± 9.8	M/F 10	M/F 9	KD: Carbohydrate: Protein: Fat = 3 ~ 6: 15 ~ 25:70 ~ 80 ketogenic ratio of 1.05 ~ 1.75:1	Carbohydrate: Protein: Fat = 55–65:7–20:15–30	3 days	Ketosis Event	occurred	not occurred	7/3	occurred	not occurred	2/7
										10 days	Ketosis Event	occurred	not occurred	5/5	occurred	not occurred	2/7

RCT: randomized controlled trial; NRCT: non randomized controlled trial; ACS: American Cancer Society diet; SD: standard diet.

### Primary patient outcomes

When the included studies categorized participants into three groups—one KD group and two control groups—we treated this as data from two distinct groups. Furthermore, when a single study reported outcomes for the KD group and the control group at different intervention durations, we considered these as separate datasets (Table 4).

**Table 4:** Study quality and risk of bias assessment using the Cochrane collaboration’s tool.

Study	Random sequence generation	Allocation concealment	Blinding	Blinding of outcome assessment	Incomplete outcome data	Selective reporting	Other bias	Score	Overall quality
Augustus et al. (33)	+	−	−	−	?	+	+	3	Fair
Budipramana et al. (35)	+	−	−	−	+	+	+	4	Good
Cohen et al. (28)	+	+	−	?	+	+	+	5	Good
Cohen et al., (32)	+	+	−	?	?	+	+	4	Good
Freedland et al. (18)	+	+	−	−	+	+	+	5	Good
Kämmerer et al. (13)	−	−	−	−	?	+	?	1	Poor
Kang et al. (31)	+	?	−	−	+	+	+	4	Good
Khodabakhshi et al. (12)	+	+	−	−	+	+	+	5	Good
Khodabakhshi et al. (12)	+	+	−	−	+	+	+	5	Good
Klement et al. (15)	−	−	−	−	+	+	+	3	Poor
Klement et al. (16)	−	−	−	−	+	+	+	3	Poor
Ok et al. (34)	+	?	−	−	+	?	−	2	Poor
Santos et al. (36)	?	?	−	?	+	+	+	3	Fair
Voss et al. (29)	+	?	−	−	+	+	+	4	Good

### Body composition

#### Fat mass

**Figure 2:**
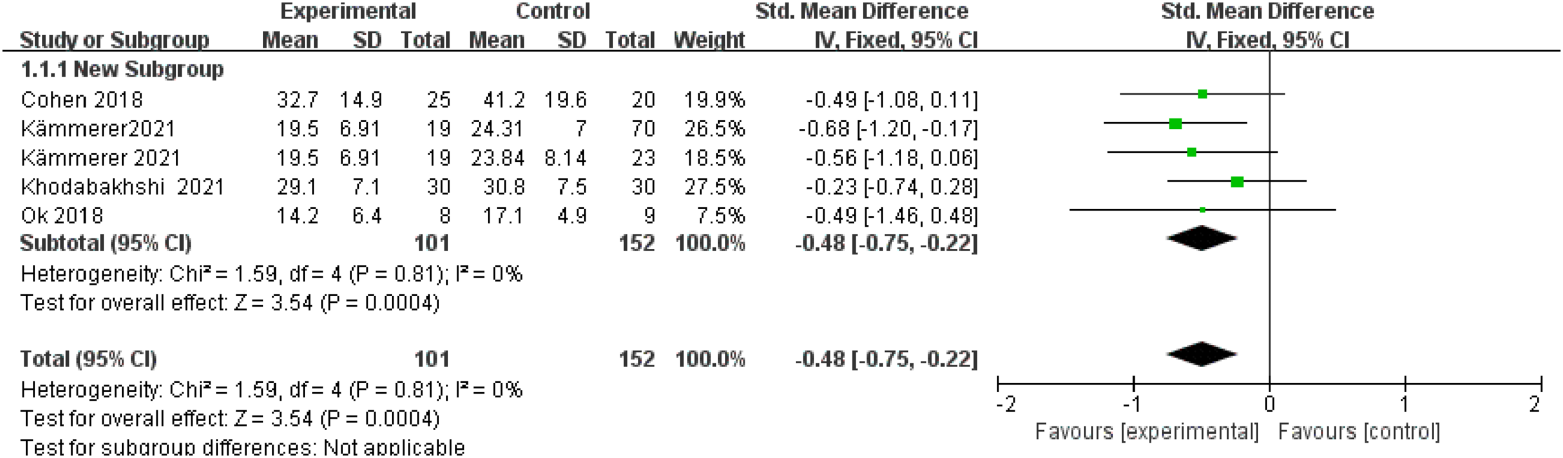
Fat mass.

Of the 14 articles included, four reported post-intervention fat mass. A fixed-effects model showed a significant reduction (SMD = −0.48; 95% CI: −0.75 to −0.22; I^2^ = 0%), indicating extremely low heterogeneity. The overall effect was statistically significant, with a *p*-value of < 0.001 (Figure 2).

#### Visceral fat mass

**Figure 3:**
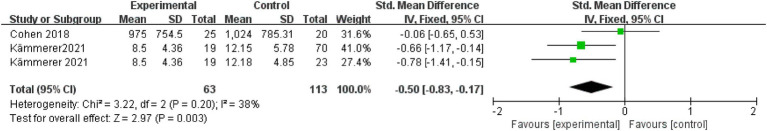
Visceral fat mass.

Two studies (with a total of 176 patients) reported that KD reduces visceral fat mass in cancer patients (SMD = −0.50, 95%CI: −0.83 to −0.17, *p* = 0.003) (Figure 3). Heterogeneity was mild (Q = 3.22, *p* = 0.200; I^2^ = 38%).

### Blood constituent

#### LDL-cholesterol

**Figure 4:**
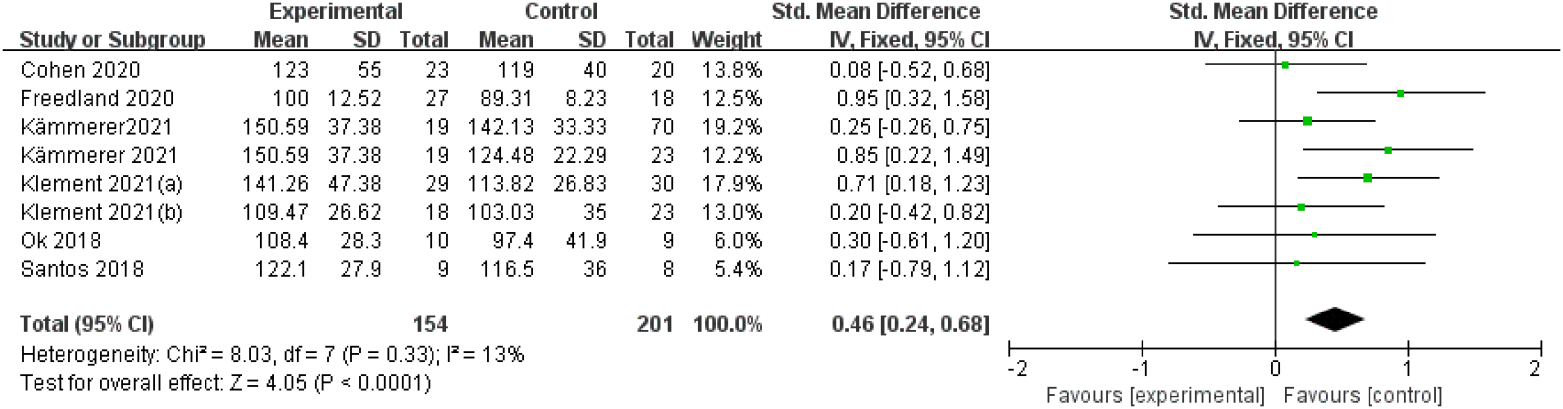
LDL-Cholesterol.

Seven articles were assessed for LDL cholesterol. A fixed-effects model was applied after the intervention to examine the change in LDL cholesterol level, with an SMD of 0.46 (95% CI: 0.24 to 0.68), I^2^ = 13%, indicating low heterogeneity. The overall effect was statistically significant, with a *p*-value of < 0.001 (Figure 4).

#### Total cholesterol (TC)

**Figure 5:**
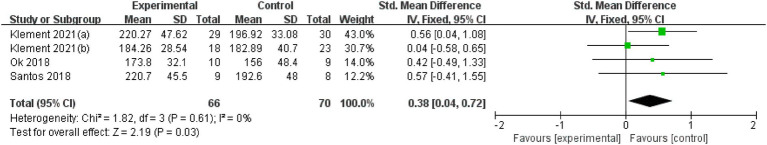
Total cholesterol (TC).

Four studies (patients’ number = 136) reported that KD reduces TC in cancer patients (SMD = 0.38, 95% CI: 0.04 to 0.72; *p* = 0.030) (Figure 5). Heterogeneity was low (Q = 1.82; *p* = 0.610; I^2^ = 0%).

### Secondary patient outcome

#### Immune-related index

##### Insulin

Eight articles assessed insulin levels. Post-intervention, a random-effects model yielded an SMD of −0.46 (95% CI: −0.85 to −0.08; I^2^ = 73%), indicating high heterogeneity. The overall effect was *p* = 0.020 (Supplementary Figure 1).

##### Blood glucose

Seven studies (involving 314 patients) reported that KD reduces blood glucose in cancer patients (SMD = −0.70, 95% CI: −1.35 to −0.05; *p* = 0.030) (Supplementary Figure 2). Heterogeneity was high (Q = 0.65; *p* < 0.001; I^2^ = 86%).

#### β-hydroxybutyrate

Four studies (with a total of 161 patients) reported that KD reduces β-hydroxybutyrate in cancer patients (SMD = 0.90, 95% CI: 0.26 to 1.54; *p* = 0.006) (Supplementary Figure 3). Heterogeneity was high (Q = 0.29; *p* = 0.020; I^2^ = 71%).

### Internal secretion

#### Thyroid-stimulating hormone (TSH)

Three studies (patients’ number = 231) reported that KD reduces TSH in cancer patients (SMD = 0.34, 95% CI: 0.06 to 0.62; *p* = 0.020) (Supplementary Figure 4). Heterogeneity was low (Q = 1.43; *p* = 0.700; I^2^ = 0%).

### Uptake

Three studies (with a total of 229 patients) reported that KD increases protein uptake in cancer patients (SMD = 4.67, 95% CI: 0.24 to 9.09; *p* < 0.001) (Supplementary Figure 5). Heterogeneity was low (Q = 19.64; *p* = 0.040; I^2^ = 0%).

### Quality of life (QOL)

#### Emotional function

Four studies (patients’ number = 299) reported that KD enhances emotional function in cancer patients (SMD = 0.37, 95% CI: 0.12 to 0.61; *p* = 0.003) (Supplementary Figure 6). Heterogeneity was low (Q = 0.28, *p* = 0.680; I^2^ = 0%).

#### Fatigue

Four studies (patients’ number = 299) reported that KD reduces fatigue in cancer patients (SMD = −0.52, 95% CI: −0.72 to −0.27; *p* < 0.001) (Supplementary Figure 7). Heterogeneity was low (Q = 7.7, *p* = 0.100; I^2^ = 48%).

#### Insomnia

Four articles were assessed for insomnia. A random-effects model was used at post-intervention for insomnia, with an SMD of −1.10 (95% CI: −2.05 to −0.15), and I^2^ = 92%, suggesting high heterogeneity. The overall effect was *p* = 0.020 (Supplementary Figure 8).

#### Social function

Three studies (*n* = 160 patients) reported that KD increases social function in cancer patients (SMD = 0.76, 95% CI: 0.14 to 1.37; *p* = 0.020) (Supplementary Figure 9). Heterogeneity was high (Q = 0.21, *p* = 0.030; I^2^ = 72%).

### Ketosis event and adverse event

#### Ketosis event

Two studies reported ketosis events, with urine testing being the most commonly used method for detecting ketone bodies. Results showed that KD had a significant effect on ketone bodies, with an odds ratio (OR) of 7.54 (95% CI, 2.57–22.13; *p* < 0.001) (Supplementary Figure 10).

### CRP subgroup analysis

#### Dietary intervention cycle

The intervention periods for the four articles were less than 6 weeks, 6–12 weeks, and more than 12 weeks, respectively. There was a statistically significant overall effect (I^2^ = 0%, *p* < 0.001), suggesting that the dietary intervention cycle had an impact on the relationship between KD and CRP outcome measures. The greatest effect size for more than 12 weeks in improving CRP outcomes was SMD = −0.63 (95% CI: −1.03 to −0.24; *p* = 0.002). For less than 6 weeks, the effect size was SMD = −0.49 (95% CI: −1.46 to 0.48; *p* = 0.330). The 6–12 week group showed the smallest effect size: SMD = −0.34 (95% CI: −0.72 to 0.05; *p* = 0.090) (Supplementary Figure 11).

### Ketogenic diet intervention ratio

#### CHO

The carbohydrate proportions reported in the four articles were 2–4%, 3–6%, 5, and 6%, respectively. The overall effect was statistically significant (I^2^ = 0%, *p* < 0.001), indicating that the CHO ratio had an effect on the relationship between KD and CRP outcomes. The greatest effect was observed in the 2–4% CHO group (SMD = −0.63; 95% CI: −1.03 to −0.24; *p* = 0.002), followed by the 3–6 and 5% CHO groups, with effect sizes of SMD = −0.49 (95% CI: −1.46 to 0.48; *p* = 0.330) and SMD = −0.49 (95% CI: −1.18 to 0.11; *p* = 0.110), respectively. The 6% CHO group showed the smallest effect size (SMD = −0.23; 95% CI: −0.74 to 0.28; *p* = 0.380) (Supplementary Figure 12).

#### Protein

The protein proportions in the four articles were 19, 15–20%, 16–18%, and 25%, respectively. The overall effect was statistically significant (I^2^ = 0%, *p* < 0.001), indicating that the protein ratio had a significant impact on the relationship between KD and CRP outcomes. The greatest effect size was observed in the 16–18% protein group (SMD = −0.63; 95% CI: −1.03 to −0.24; *p* = 0.0020), followed by the 15–20 and 25% protein groups, with effect sizes of SMD = −0.49 (95% CI: −1.46 to 0.48; *p* = 0.330) and SMD = −0.49 (95% CI: −1.08 to 0.11; *p* = 0.110), respectively. The 19% protein group showed the smallest effect size (SMD = −0.23; 95% CI: −0.74 to 0.28; *p* = 0.380) (Supplementary Figure 13).

#### Fat

The proportions of fat reported in the four articles were 55, 70%, 70–80, and 80–85%, respectively. The overall effect was statistically significant (I^2^ = 0%, *p* < 0.001), indicating that the fat ratio had a significant impact on the relationship between KD and CRP outcomes. The greatest effect size was observed in the 80–85% fat group (SMD = −0.63; 95% CI: −1.03 to −0.24; *p* = 0.002), followed by the 70–80 and 70% fat groups, with effect sizes of SMD = -0.49 (95% CI: −1.46 to 0.48; *p* = 0.330) and SMD = −0.49 (95% CI: −1.08 to 0.11; *p* = 0.110), respectively. The 55% fat group showed the smallest effect size (SMD = −0.23; 95% CI: −0.74 to 0.28; *p* = 0.380) (Supplementary Figure 14).

The implementation of a KD intervention in cancer patients did not result in significant changes in the following nine indicators: HDL cholesterol, triglycerides, CRP, IGF-1, TNF-*α*, creatinine, urea, energy intake, and age at the time of the dietary intervention (see Supplementary Figures 15–23).

### Publication bias and sensitivity analysis

Funnel plot analysis revealed some asymmetry in the distribution of study sites, suggesting the possibility of publication bias. A sensitivity analysis was conducted by modifying the pooling model and comparing the results after sequentially removing each article. It was found that the combined results did not change significantly, indicating that our results were stable (Supplementary Figure 24).

## Discussion

This systematic review found that the KD intervention has the potential to benefit cancer-related outcomes such as fat mass, visceral fat mass, LDL cholesterol, total cholesterol, thyroid-stimulating hormone, insulin, blood glucose, β-hydroxybutyrate, emotional function, fatigue, insomnia, social function, and ketosis events in cancer patients. It is a low-cost, easy-to-implement dietary intervention that should be recommended.

Evidence from this review suggests that KD can lead to a notable reduction in both fat mass and visceral fat mass, consistent with the findings of a previous study (37). Increased body fat mass can lead to chronic inflammation, which may further promote cancer development (38, 39). The KD, characterized by very low carbohydrate intake and high fat intake, encourages the body to convert fat into ketone bodies, which serve as the primary energy source (40). As body fat is utilized and consumed, body fat mass decreases. The KD, known for significantly reducing body fat mass, has been shown to exert anti-inflammatory, antiangiogenic, and pro-apoptotic effects on breast cancer cells (41). Evidence suggests that obesity and a surplus of adipose tissue support cancer growth through immune dysregulation, chronic inflammation, and increased insulin signaling, thereby reinforcing the causal link (41, 42). Adipose tissue, particularly around visceral organs, and its immune cells release pro-inflammatory cytokines such as interleukins and TNF-α, contributing to insulin resistance and tumor growth (43). Thus, the KD’s impact on reducing body weight and fat mass in cancer patients is beneficial. Additionally, this dietary pattern may alter the body’s metabolism to further reduce fat. However, future well-designed randomized controlled trials with broader populations are necessary to validate our findings and ultimately establish the KD as a routine adjunctive treatment for cancer patients.

Secondly, in terms of blood parameters, except for a significant decrease in low-density lipoprotein (LDL) and total cholesterol, no other outcome indicators showed significant changes. This finding is inconsistent with some previous studies and may be related to differences in intervention durations and disease types (44). In addition, the lack of significant effects for part indicators could also be attributed to the limited number of included studies and the presence of potential bias in methodological quality, as assessed by the risk of bias evaluation. Such biases may have weakened the statistical power and obscured potential associations. It is also worth noting that even in the absence of statistically significant differences, some observed trends may still hold potential clinical relevance. Future studies should consider both statistical and clinical significance when evaluating the impact of KD on metabolic and inflammatory outcomes.

Third, this review indicated that KD intervention was associated with significant improvements in emotional function, insomnia, social function, and fatigue among cancer patients; however, no significant effects were observed in other QoL domains. The improvements in emotional and social functioning align with findings from previous studies; however, the effects on fatigue and insomnia remain inconsistent across these studies (45). One possible explanation is that comprehensive improvements in QoL may require sustained adherence to the KD over a longer period. Most of the included studies only assessed short-term interventions, which may not have been sufficient to produce meaningful changes in overall QoL for patients. Therefore, further well-designed randomized controlled trials with longer follow-up durations are warranted to establish more definitive conclusions.

Furthermore, the results of this study indicate that changes in urea, creatinine, and adverse events were not significantly different, suggesting that a well-designed KD may reduce fat mass in cancer patients without causing serious adverse effects on liver and kidney function (12, 46). Therefore, we can preliminarily conclude that the studies included in this review do not show any detrimental effects of the KD on cancer patients, and it appears to have a certain level of safety.

Khodabakhshi et al. (47) suggest that the positive effects of ketogenic diet intervention on cancer patients are closely related to the duration of the intervention. The most significant finding of this study is that cancer patients experience the greatest improvement in C-reactive protein levels when following a KD for a duration of more than 12 weeks. In addition, it is pointed out that the proportion of KD gradients has a positive and significant effect on cancer patients (48). The results of this study indicate that 2–4% carbohydrates, 16–18% protein, and 80–85% fat are the maximum effective amounts for improving cancer patients’ outcomes. This provides precise recommendations for the design of future clinical trials. At present, there are significant differences in dietary recommendations, further highlighting the importance of evidence-based guidelines for ketogenic dietary intervention research (49). Currently, the implementation of KD interventions in clinical settings often lacks standardized guidelines and consistent food sources, which may affect the accuracy and consistency of the outcomes. The results of this study offer evidence that can help standardize KD interventions.

Finally, similar to earlier meta-analysis findings, our results demonstrate generally positive effects of KDs on weight loss or maintenance (50–52). Moreover, potential benefits on glycemic control and lipid regulation have also been consistently reported across studies (37, 45, 50). Importantly, this study addresses a critical gap in the current literature on precision nutritional interventions by stratifying outcomes based on cancer types, treatment stages, and dietary strategies. It further contributes novel insights into the application of synchronized intervention timing and dynamic monitoring of safety indicators. Nevertheless, future research with larger sample sizes and extended follow-up durations is necessary to confirm the long-term benefits of ketogenic dietary interventions.

## Limitations

This study has several limitations. First, although the included studies were from different countries worldwide, there was limited evidence from regions such as Africa and Asia, which may have introduced regional bias. Second, the methodological quality of most included studies was low, resulting in a limited level of evidence. Third, the types, stages, and treatment methods of cancer included in this study were diverse, and the specific content of the KD interventions and outcome measurement standards varied, which supports the notion that obesity and a surplus of adipose tissue are not uniform, potentially increasing the heterogeneity of the results. Fourth, most studies only reported the short-term effects of KD interventions, lacking verification of long-term effects. Therefore, the conclusions drawn from this study require longer intervention periods to be substantiated. In conclusion, due to the small amount of evidence, the mixture of RCTs and non-RCTs, the inclusion of non-RCTs, and the small sample size, this may lead to high heterogeneity, which in turn may not lead to a definitive causality. The results derived from the present study represent only a preliminary exploration for the validation of the relevant results at a later stage. Finally, publication bias may have influenced the results of this review, as suggested by asymmetry in the funnel plot. This could be related to the limited number of studies, selective reporting of positive findings, and potential language or regional publication restrictions. Therefore, the findings should be interpreted with caution.

### Implications for future studies

Despite the limitations of this meta-analysis, it may still offer valuable insights for future randomized controlled trials of KD interventions. Researchers should focus on methodological quality, employing rigorous and blinded study designs, and conducting large-sample, multicenter studies in specific cancer populations to enhance the level of evidence and verify the effectiveness and safety of KD interventions in cancer patients. Additionally, outcome measures should combine both subjective and objective indicators to establish the scientific validity of the conclusions from multiple perspectives. The KD intervention should be more standardized. For example, the KD intervention cycle, specific types of intervention diet, intervention frequency, single intervention time, intervention intensity, and other effects. Furthermore, researchers should conduct follow-up studies to observe the long-term effects of KD intervention, providing a more reliable basis for the treatment and care of cancer patients. This study suggests that future studies of the KD should enhance sample size, follow-up time, control group settings, and nutritional monitoring to further validate the reliability of the results derived in this study and to make a greater contribution to enriching the benefits of the KD for cancer patients.

## Conclusion

This review found that KD results in improved cancer-related fat mass, visceral fat mass, LDL cholesterol, total cholesterol, β-hydroxybutyrate, thyroid-stimulating hormone, insulin, blood glucose, emotional function, fatigue, insomnia, social function, and ketosis events. Furthermore, the C-reactive protein outcome index had a greater impact when the intervention period exceeded 12 weeks, with a proportion of 2–4% CHO, 16–18% protein, and 80–85% fat. The potential benefits of a KD in cancer treatment highlight the necessity for well-designed clinical trials to better understand how this adjunctive approach impacts cancer patients’ nutritional status, prognosis, and QoL.

## Data Availability

The original contributions presented in the study are included in the article/Supplementary material, further inquiries can be directed to the corresponding author.
