# Supplementary material for: Impact of ketogenic diets on cancer patient outcomes: a systematic review and meta-analysis
Source: Front Nutr. 2025 Jul 18;12:1535921. doi: 10.3389/fnut.2025.1535921 (PMC12313497; doi:10.3389/fnut.2025.1535921)

Supplementary Figures

Supplementary Figure 1. Insulin

Study or Subgroup	Experimental Mean	SD	Total	Control Mean	SD	Total	Weight	Std. Mean Difference IV, Random, 95% CI
Cohen 2018	6.7	4.1	23	12.1	6.7	20	11.0%	-0.97 [-1.61, -0.33]
Kämmerer2021	12.45	6.85	19	14.7	8.43	70	12.3%	-0.27 [-0.78, 0.23]
Kämmerer 2021	12.45	6.85	19	15.82	9.25	23	11.2%	-0.40 [-1.01, 0.21]
Kang 2019	11.58	10.52	9	6.14	2.63	9	8.0%	0.68 [-0.28, 1.63]
Khodabakhshi 2020(a)	6.1	6.7	30	9.1	9.6	30	12.3%	-0.36 [-0.87, 0.15]
Khodabakhshi 2021	5.7	4	30	6.9	4.5	30	12.3%	-0.28 [-0.79, 0.23]
Klement 2021(a)	9.29	7.6	29	8.35	4.97	30	12.3%	0.14 [-0.37, 0.66]
Klement 2021(b)	0.89	5.52	18	20.45	12.5	23	9.8%	-1.90 [-2.66, -1.15]
Ok 2018	0	0	0	0	0	0		Not estimable
Voss 2020	6.9	3.7	22	17.25	19.17	18	10.9%	-0.77 [-1.42, -0.13]
**Total (95% CI)**			**199**			**253**	**100.0%**	**-0.46 [-0.85, -0.08]**

Heterogeneity: Tau² = 0.25; Chi² = 29.31, df = 8 (P = 0.0003); I² = 73%
Test for overall effect: Z = 2.35 (P = 0.02)

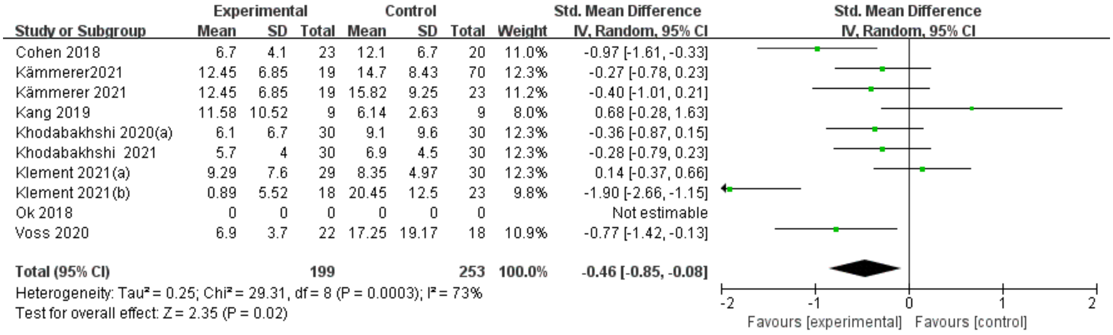

Supplementary Figure 2. Blood glucose

Study or Subgroup	Experimental Mean	SD	Total	Control Mean	SD	Total	Weight	Std. Mean Difference IV, Random, 95% CI
Augustus 2020	74.8	6.204	17	84.4	7.358	20	13.9%	-1.37 [-2.10, -0.64]
Cohen 2018	93	15.83	23	98.75	11.8	20	14.7%	-0.40 [-1.01, 0.21]
Freedland 2020	98.63	3.76	27	106.53	5.21	18	14.0%	-1.77 [-2.48, -1.06]
Kang 2019	132.14	246.43	9	106.25	77.68	9	12.5%	0.13 [-0.79, 1.06]
Khodabakhshi 2019	84.5	11.3	30	105.2	15.8	30	14.8%	-1.49 [-2.06, -0.91]
Klement 2021(a)	103.15	16.29	29	96.76	9.31	30	15.2%	0.48 [-0.04, 1.00]
Klement 2021(b)	108.73	22.51	29	131.27	61.96	23	15.0%	-0.50 [-1.06, 0.06]
**Total (95% CI)**			**164**			**150**	**100.0%**	**-0.70 [-1.35, -0.05]**

Heterogeneity: Tau² = 0.65; Chi² = 43.34, df = 6 (P < 0.00001); I² = 86%
Test for overall effect: Z = 2.12 (P = 0.03)

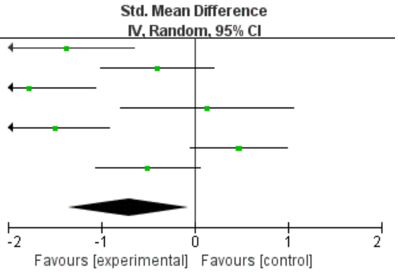

Supplementary Figure 3. β-hydroxybutyrate

Study or Subgroup	Experimental Mean	SD	Total	Control Mean	SD	Total	Weight	Std. Mean Difference IV, Random, 95% CI
Cohen 2018	0.91	0.73	23	0.25	0.18	20	26.1%	1.18 [0.53, 1.83]
Kang 2019	0.29	0.17	9	0.27	0.15	9	20.5%	0.12 [-0.81, 1.04]
Klement 2021(a)	0.67	0.55	29	0.36	0.63	30	29.0%	0.52 [-0.00, 1.04]
Klement 2021(b)	0.86	0.61	18	0.14	0.12	23	24.4%	1.71 [0.98, 2.44]
**Total (95% CI)**			**79**			**82**	**100.0%**	**0.90 [0.26, 1.54]**

Heterogeneity: Tau² = 0.29; Chi² = 10.28, df = 3 (P = 0.02); I² = 71%
Test for overall effect: Z = 2.77 (P = 0.006)

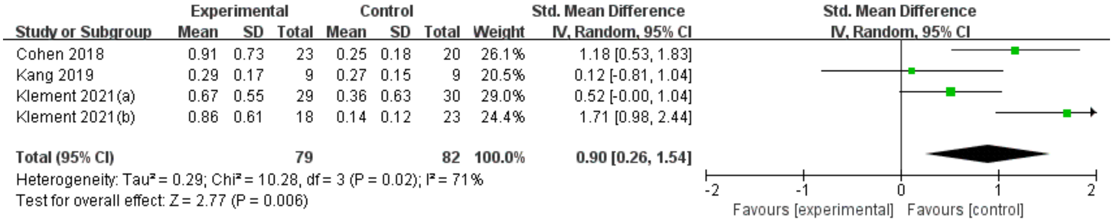

Supplementary Figure 4. Thyroid stimulating hormone (TSH)

Study or Subgroup	Experimental Mean	SD	Total	Control Mean	SD	Total	Weight	Std. Mean Difference IV, Fixed, 95% CI
Kämmerer2021	1.83	1.3	19	1.61	0.94	70	30.2%	0.21 [-0.30, 0.72]
Kämmerer 2021	1.83	1.3	19	1.2	0.88	23	20.2%	0.57 [-0.05, 1.19]
Klement 2021(a)	1.76	0.97	29	1.6	0.74	30	29.8%	0.18 [-0.33, 0.69]
Klement 2021(b)	6.21	9.17	18	2.79	3	23	19.8%	0.52 [-0.11, 1.15]
**Total (95% CI)**			**85**			**146**	**100.0%**	**0.34 [0.06, 0.62]**

Heterogeneity: Chi² = 1.43, df = 3 (P = 0.70); I² = 0%
Test for overall effect: Z = 2.36 (P = 0.02)

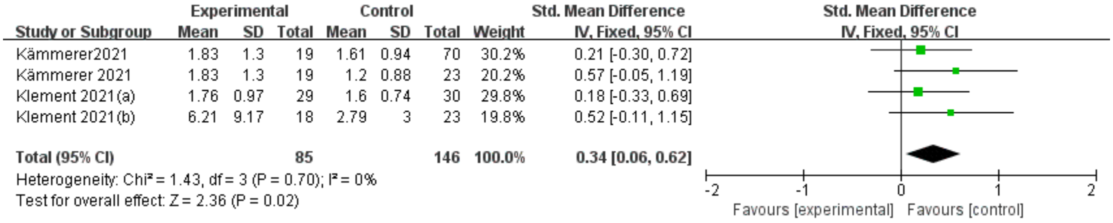

Supplementary Figure 5. Protein uptake

Study or Subgroup	Experimental Mean	Experimental SD	Experimental Total	Control Mean	Control SD	Control Total	Weight	Std. Mean Difference IV, Random, 95% CI
Cohen 2020	29	0.7	23	18.5	1	15	23.2%	12.39 [9.38, 15.40]
Kämmerer2021	1.33	0.07	19	1.2	0.03	70	25.8%	3.10 [2.41, 3.79]
Kämmerer 2021	1.33	0.07	19	0.98	0.04	23	25.2%	6.18 [4.67, 7.70]
Khodabakhshi 2020(a)	57.12	5.15	30	69.95	6.37	30	25.8%	-2.19 [-2.83, -1.54]
**Total (95% CI)**			**91**			**138**	**100.0%**	**4.67 [0.24, 9.09]**

Heterogeneity: Tau² = 19.64; Chi² = 228.32, df = 3 (P < 0.00001); I² = 99%
Test for overall effect: Z = 2.07 (P = 0.04)

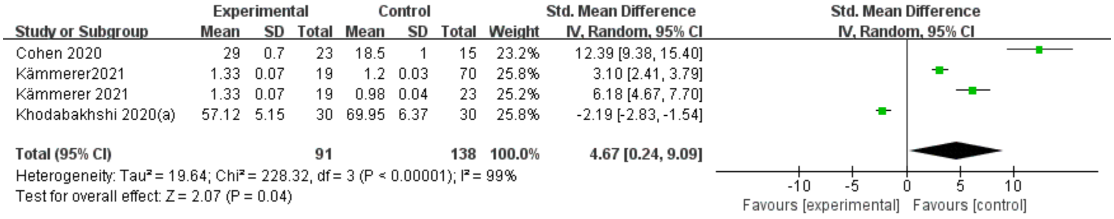

Supplementary Figure 6. Emotional function

Study or Subgroup	Experimental Mean	Experimental SD	Experimental Total	Control Mean	Control SD	Control Total	Weight	Std. Mean Difference IV, Fixed, 95% CI
Kämmerer2021	70.52	26.76	20	64.43	20.88	75	24.1%	0.27 [-0.22, 0.77]
Kämmerer 2021	70.52	26.76	20	56.06	25.68	24	16.1%	0.54 [-0.06, 1.15]
Khodabakhshi 2020(a)	62	23	30	60	21	30	23.0%	0.09 [-0.42, 0.60]
Klement 2021(a)	75	12.34	29	66.69	16.35	30	21.7%	0.56 [0.04, 1.09]
Klement 2021(b)	74.2	18.57	18	64.28	22.04	23	15.1%	0.47 [-0.15, 1.10]
**Total (95% CI)**			**117**			**182**	**100.0%**	**0.37 [0.12, 0.61]**

Heterogeneity: Chi² = 2.28, df = 4 (P = 0.68); I² = 0%
Test for overall effect: Z = 2.96 (P = 0.003)

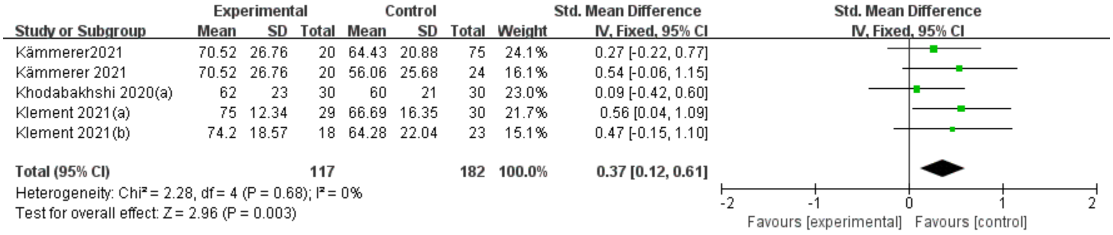

Supplementary Figure 7. Fatigue

Study or Subgroup	Experimental Mean	Experimental SD	Experimental Total	Control Mean	Control SD	Control Total	Weight	Std. Mean Difference IV, Fixed, 95% CI
Kämmerer2021	21.01	23.79	20	35.57	20.88	75	23.9%	-0.67 [-1.17, -0.17]
Kämmerer 2021	21.01	23.79	20	48.5	22.83	24	14.5%	-1.16 [-1.81, -0.51]
Khodabakhshi 2020(a)	33.95	8.82	30	35.62	5.39	30	23.4%	-0.23 [-0.73, 0.28]
Klement 2021(a)	34.66	19.19	29	44.18	13.63	30	22.2%	-0.57 [-1.09, -0.04]
Klement 2021(b)	36.9	24.75	18	38.14	26.46	23	15.9%	-0.05 [-0.66, 0.57]
**Total (95% CI)**			**117**			**182**	**100.0%**	**-0.52 [-0.76, -0.27]**

Heterogeneity: Chi² = 7.70, df = 4 (P = 0.10); I² = 48%
Test for overall effect: Z = 4.11 (P < 0.0001)

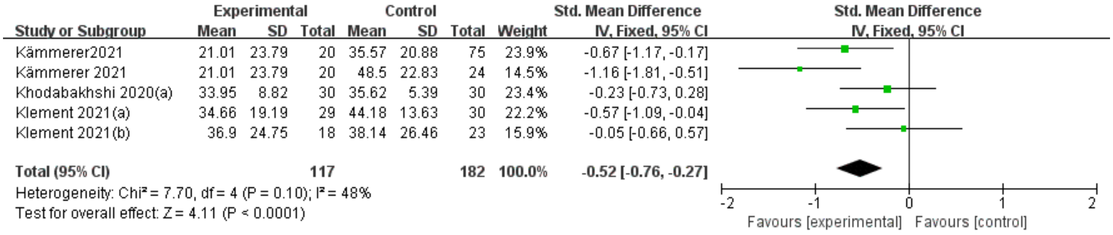

Supplementary Figure 8. Insomnia

Study or Subgroup	Experimental Mean	Experimental SD	Experimental Total	Control Mean	Control SD	Control Total	Weight	Std. Mean Difference IV, Random, 95% CI
Kämmerer2021	38.26	26.76	20	64.43	20.88	75	20.5%	-1.17 [-1.69, -0.65]
Kämmerer 2021	38.26	26.76	20	37.8	25.68	24	20.1%	0.02 [-0.58, 0.61]
Khodabakhshi 2020(a)	4.88	8.88	30	31.1	12.25	30	19.7%	-2.42 [-3.09, -1.74]
Klement 2021(a)	37.35	24.68	29	37.27	24.51	30	20.6%	0.00 [-0.51, 0.51]
Klement 2021(b)	33.32	18.57	18	70.78	17.81	23	19.1%	-2.02 [-2.79, -1.25]
**Total (95% CI)**			**117**			**182**	**100.0%**	**-1.10 [-2.05, -0.15]**

Heterogeneity: Tau² = 1.07; Chi² = 49.83, df = 4 (P < 0.00001); I² = 92%
Test for overall effect: Z = 2.27 (P = 0.02)

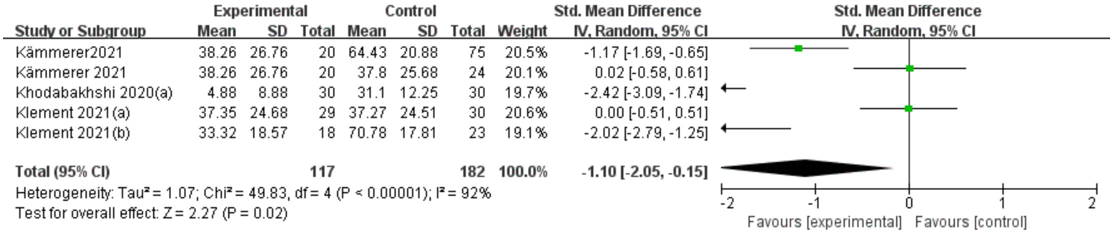

Supplementary Figure 9. Social function

Study or Subgroup	Experimental Mean	Experimental SD	Experimental Total	Control Mean	Control SD	Control Total	Weight	Std. Mean Difference IV, Random, 95% CI
Khodabakhshi 2020(a)	91	17	30	87	17	30	35.6%	0.23 [-0.28, 0.74]
Klement 2021(a)	79.27	16.45	29	66.69	16.35	30	34.9%	0.76 [0.23, 1.29]
Klement 2021(b)	83.32	9.27	18	53.98	26.46	23	29.5%	1.38 [0.69, 2.08]
**Total (95% CI)**			**77**			**83**	**100.0%**	**0.76 [0.14, 1.37]**

Heterogeneity: Tau² = 0.21; Chi² = 7.03, df = 2 (P = 0.03); I² = 72%
Test for overall effect: Z = 2.39 (P = 0.02)

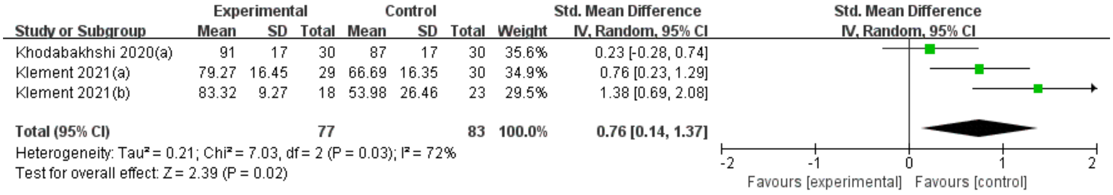

Supplementary Figure 10. Ketosis Event (Odds Ratio)

Study or Subgroup	Experimental Events	Experimental Total	Control Events	Control Total	Weight	Odds Ratio M-H, Fixed, 95% CI
Cohen 2018	13	23	2	20	35.6%	11.70 [2.19, 62.62]
Ok2018	7	10	2	9	24.2%	8.17 [1.03, 64.94]
Ok 2018	5	10	2	9	40.3%	3.50 [0.47, 25.90]
**Total (95% CI)**		**43**		**38**	**100.0%**	**7.54 [2.57, 22.13]**
Total events	25		6			

Heterogeneity: Chi² = 0.83, df = 2 (P = 0.66); I² = 0%
Test for overall effect: Z = 3.68 (P = 0.0002)

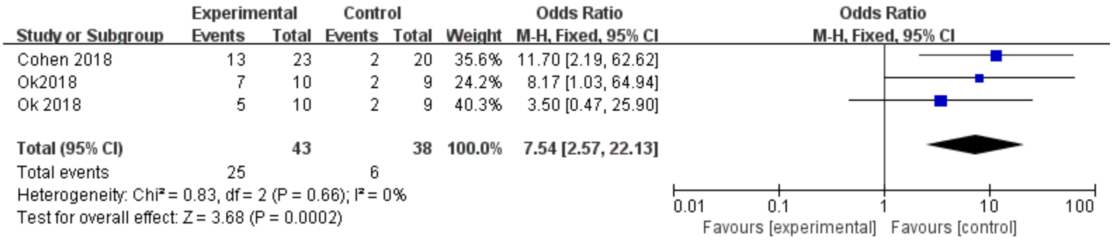

Supplementary Figure 11. Dietary intervention cycle

Study or Subgroup	Experimental Mean	Experimental SD	Experimental Total	Control Mean	Control SD	Control Total	Weight	Std. Mean Difference IV, Fixed, 95% CI
**6.2.1 <6 weeks**								
Ok 2018	14.2	6.4	8	17.1	4.9	9	7.5%	-0.49 [-1.46, 0.48]
**Subtotal (95% CI)**			**8**			**9**	**7.5%**	**-0.49 [-1.46, 0.48]**
Heterogeneity: Not applicable								
Test for overall effect: Z = 0.98 (P = 0.33)								
**6.2.2 6-12 weeks**								
Cohen 2018	32.7	14.9	25	41.2	19.6	20	19.9%	-0.49 [-1.08, 0.11]
Khodabakhshi 2021	29.1	7.1	30	30.8	7.5	30	27.5%	-0.23 [-0.74, 0.28]
**Subtotal (95% CI)**			**55**			**50**	**47.5%**	**-0.34 [-0.72, 0.05]**
Heterogeneity: Chi² = 0.41, df = 1 (P = 0.52); I² = 0%								
Test for overall effect: Z = 1.71 (P = 0.09)								
**6.2.3 >12 weeks**								
Kämmerer2021	19.5	6.91	19	24.31	7	70	26.5%	-0.68 [-1.20, -0.17]
Kämmerer 2021	19.5	6.91	19	23.84	8.14	23	18.5%	-0.56 [-1.18, 0.06]
**Subtotal (95% CI)**			**38**			**93**	**45.0%**	**-0.63 [-1.03, -0.24]**
Heterogeneity: Chi² = 0.09, df = 1 (P = 0.76); I² = 0%								
Test for overall effect: Z = 3.12 (P = 0.002)								
**Total (95% CI)**			**101**			**152**	**100.0%**	**-0.48 [-0.75, -0.22]**

Heterogeneity: Chi² = 1.59, df = 4 (P = 0.81); I² = 0%
Test for overall effect: Z = 3.54 (P = 0.0004)
Test for subgroup differences: Chi² = 1.08, df = 2 (P = 0.58), I² = 0%

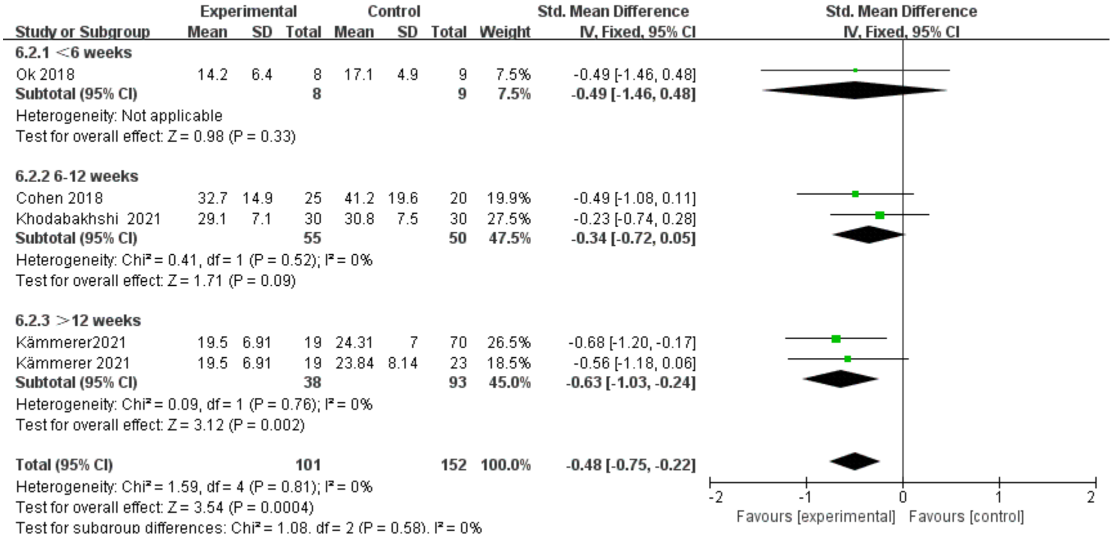

Supplementary Figure 12. Dietary intervention CHO ratio

Study or Subgroup	Experimental Mean	Experimental SD	Experimental Total	Control Mean	Control SD	Control Total	Weight	Std. Mean Difference IV, Fixed, 95% CI
**6.3.1 2%-4% CHO**								
Kämmerer2021	19.5	6.91	19	24.31	7	70	26.5%	-0.68 [-1.20, -0.17]
Kämmerer 2021	19.5	6.91	19	23.84	8.14	23	18.5%	-0.56 [-1.18, 0.06]
**Subtotal (95% CI)**			**38**			**93**	**45.0%**	**-0.63 [-1.03, -0.24]**
Heterogeneity: Chi² = 0.09, df = 1 (P = 0.76); I² = 0%								
Test for overall effect: Z = 3.12 (P = 0.002)								
**6.3.2 3%-6% CHO**								
Ok 2018	14.2	6.4	8	17.1	4.9	9	7.5%	-0.49 [-1.46, 0.48]
**Subtotal (95% CI)**			**8**			**9**	**7.5%**	**-0.49 [-1.46, 0.48]**
Heterogeneity: Not applicable								
Test for overall effect: Z = 0.98 (P = 0.33)								
**6.3.3 5% CHO**								
Cohen 2018	32.7	14.9	25	41.2	19.6	20	19.9%	-0.49 [-1.08, 0.11]
**Subtotal (95% CI)**			**25**			**20**	**19.9%**	**-0.49 [-1.08, 0.11]**
Heterogeneity: Not applicable								
Test for overall effect: Z = 1.60 (P = 0.11)								
**6.3.4 6% CHO**								
Khodabakhshi 2021	29.1	7.1	30	30.8	7.5	30	27.5%	-0.23 [-0.74, 0.28]
**Subtotal (95% CI)**			**30**			**30**	**27.5%**	**-0.23 [-0.74, 0.28]**
Heterogeneity: Not applicable								
Test for overall effect: Z = 0.89 (P = 0.38)								
**Total (95% CI)**			**101**			**152**	**100.0%**	**-0.48 [-0.75, -0.22]**
Heterogeneity: Chi² = 1.59, df = 4 (P = 0.81); I² = 0%								
Test for overall effect: Z = 3.54 (P = 0.0004)								
Test for subgroup differences: Chi² = 1.50, df = 3 (P = 0.68), I² = 0%								

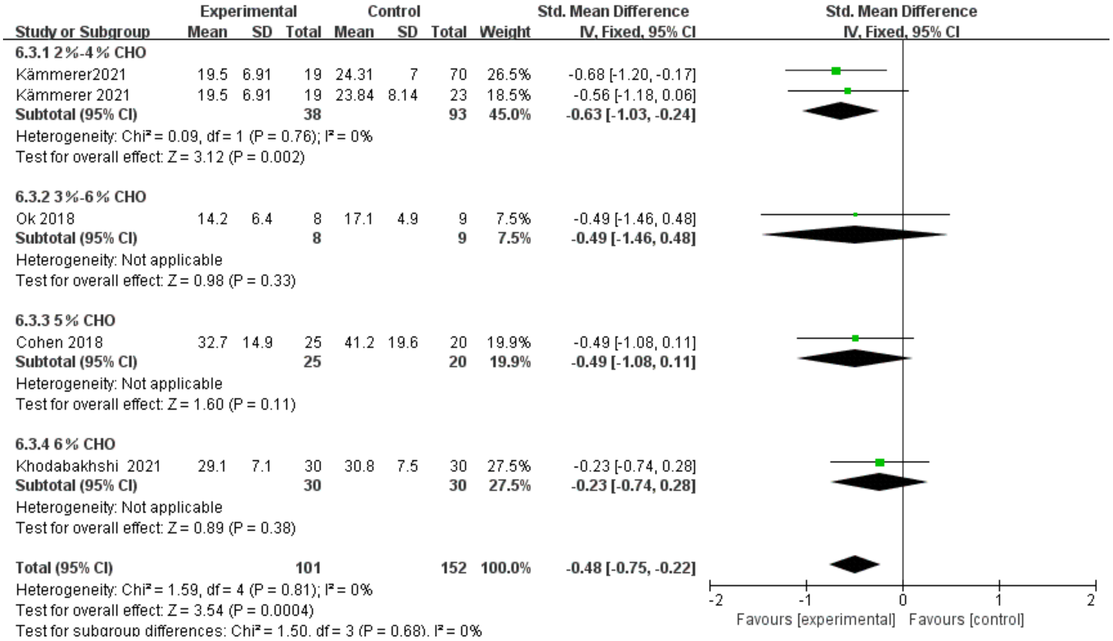

Supplementary Figure 13. Dietary intervention protein ratio

Study or Subgroup	Experimental Mean	Experimental SD	Experimental Total	Control Mean	Control SD	Control Total	Weight	Std. Mean Difference IV, Fixed, 95% CI
**6.4.1 15%-25% Protein**								
Ok 2018	14.2	6.4	8	17.1	4.9	9	7.5%	-0.49 [-1.46, 0.48]
**Subtotal (95% CI)**			**8**			**9**	**7.5%**	**-0.49 [-1.46, 0.48]**
Heterogeneity: Not applicable								
Test for overall effect: Z = 0.98 (P = 0.33)								
**6.4.2 16%-18% Protein**								
Kämmerer2021	19.5	6.91	19	24.31	7	70	26.5%	-0.68 [-1.20, -0.17]
Kämmerer 2021	19.5	6.91	19	23.84	8.14	23	18.5%	-0.56 [-1.18, 0.06]
**Subtotal (95% CI)**			**38**			**93**	**45.0%**	**-0.63 [-1.03, -0.24]**
Heterogeneity: Chi² = 0.09, df = 1 (P = 0.76); I² = 0%								
Test for overall effect: Z = 3.12 (P = 0.002)								
**6.4.3 19% Protein**								
Khodabakhshi 2021	29.1	7.1	30	30.8	7.5	30	27.5%	-0.23 [-0.74, 0.28]
**Subtotal (95% CI)**			**30**			**30**	**27.5%**	**-0.23 [-0.74, 0.28]**
Heterogeneity: Not applicable								
Test for overall effect: Z = 0.89 (P = 0.38)								
**6.4.4 25% Protein**								
Cohen 2018	32.7	14.9	25	41.2	19.6	20	19.9%	-0.49 [-1.08, 0.11]
**Subtotal (95% CI)**			**25**			**20**	**19.9%**	**-0.49 [-1.08, 0.11]**
Heterogeneity: Not applicable								
Test for overall effect: Z = 1.60 (P = 0.11)								
**Total (95% CI)**			**101**			**152**	**100.0%**	**-0.48 [-0.75, -0.22]**
Heterogeneity: Chi² = 1.59, df = 4 (P = 0.81); I² = 0%								
Test for overall effect: Z = 3.54 (P = 0.0004)								
Test for subgroup differences: Chi² = 1.50, df = 3 (P = 0.68), I² = 0%								

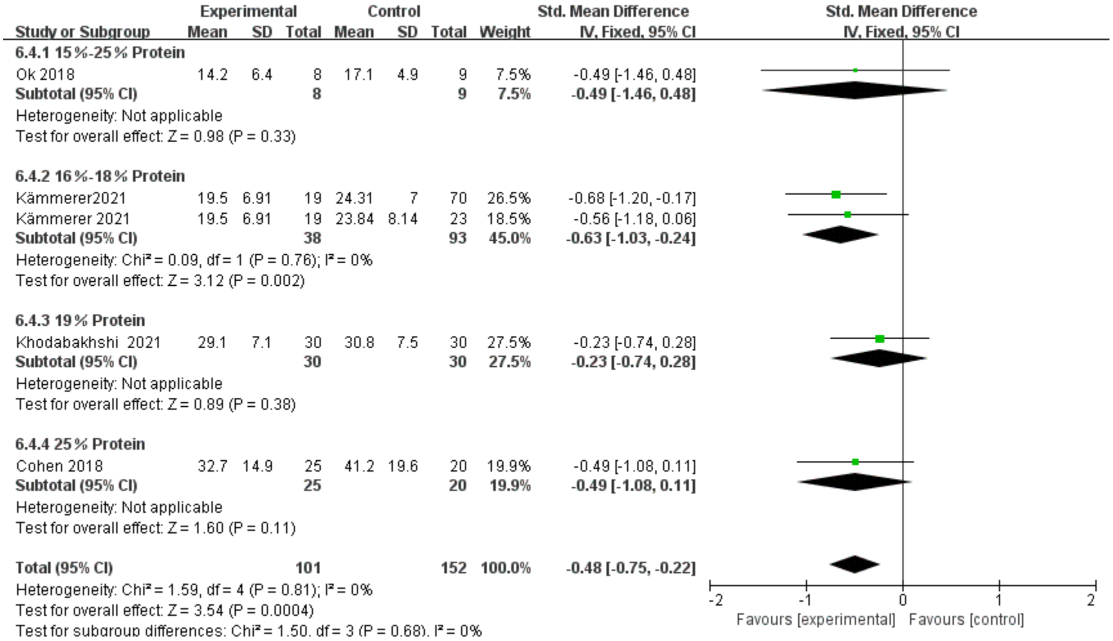

Supplementary Figure 14. Dietary intervention fat ratio

Study or Subgroup	Experimental Mean	Experimental SD	Experimental Total	Control Mean	Control SD	Control Total	Weight	Std. Mean Difference IV, Fixed, 95% CI
**6.5.1 55% Fat**								
Khodabakhshi 2021	29.1	7.1	30	30.8	7.5	30	27.5%	-0.23 [-0.74, 0.28]
**Subtotal (95% CI)**			**30**			**30**	**27.5%**	**-0.23 [-0.74, 0.28]**
Heterogeneity: Not applicable								
Test for overall effect: Z = 0.89 (P = 0.38)								
**6.5.2 70% Fat**								
Cohen 2018	32.7	14.9	25	41.2	19.6	20	19.9%	-0.49 [-1.08, 0.11]
**Subtotal (95% CI)**			**25**			**20**	**19.9%**	**-0.49 [-1.08, 0.11]**
Heterogeneity: Not applicable								
Test for overall effect: Z = 1.60 (P = 0.11)								
**6.5.3 70%-80% Fat**								
Ok 2018	14.2	6.4	8	17.1	4.9	9	7.5%	-0.49 [-1.46, 0.48]
**Subtotal (95% CI)**			**8**			**9**	**7.5%**	**-0.49 [-1.46, 0.48]**
Heterogeneity: Not applicable								
Test for overall effect: Z = 0.98 (P = 0.33)								
**6.5.4 80%-85% Fat**								
Kämmerer2021	19.5	6.91	19	24.31	7	70	26.5%	-0.68 [-1.20, -0.17]
Kämmerer 2021	19.5	6.91	19	23.84	8.14	23	18.5%	-0.56 [-1.18, 0.06]
**Subtotal (95% CI)**			**38**			**93**	**45.0%**	**-0.63 [-1.03, -0.24]**
Heterogeneity: Chi² = 0.09, df = 1 (P = 0.76); I² = 0%								
Test for overall effect: Z = 3.12 (P = 0.002)								
**Total (95% CI)**			**101**			**152**	**100.0%**	**-0.48 [-0.75, -0.22]**
Heterogeneity: Chi² = 1.59, df = 4 (P = 0.81); I² = 0%								
Test for overall effect: Z = 3.54 (P = 0.0004)								
Test for subgroup differences: Chi² = 1.50, df = 3 (P = 0.68), I² = 0%								

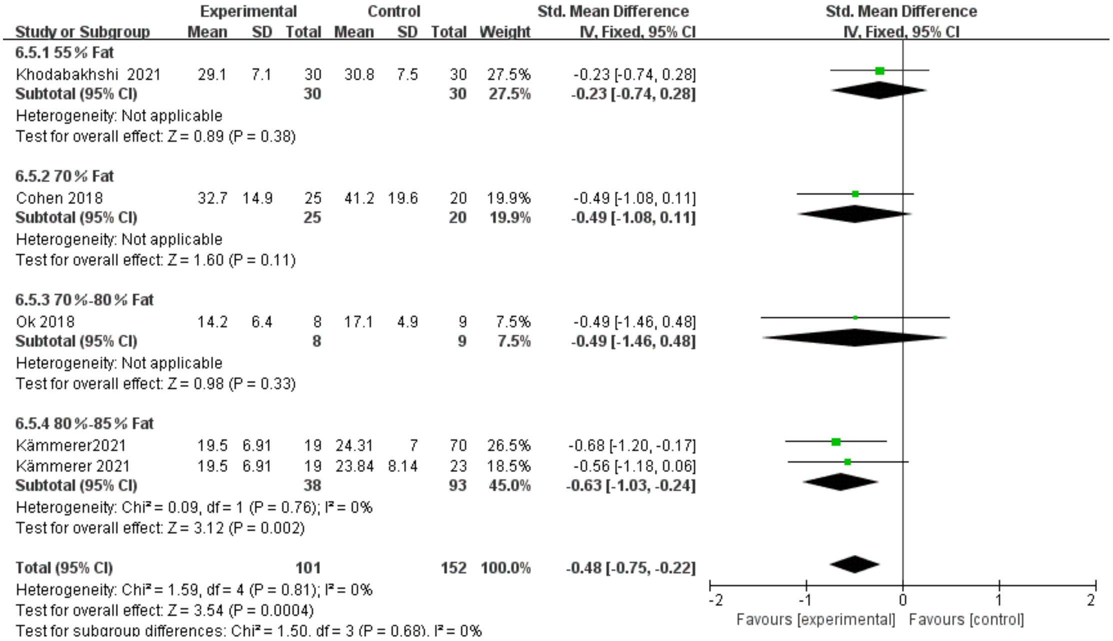

Supplementary Figure 15. HDL-Cholesterol

Study or Subgroup	Experimental Mean	Experimental SD	Experimental Total	Control Mean	Control SD	Control Total	Weight	Std. Mean Difference IV, Random, 95% CI
Cohen 2020	72	13	23	65	15	20	14.5%	0.49 [-0.12, 1.10]
Freedland 2020	46.5	3	27	49.87	6.31	18	14.4%	-0.72 [-1.34, -0.10]
Kämmerer2021	75.83	22.21	19	67.64	13.71	70	16.5%	0.51 [-0.00, 1.03]
Kämmerer 2021	75.83	22.21	19	65.38	12.96	23	14.3%	0.58 [-0.04, 1.20]
Klement 2021(a)	73.06	24.18	29	67.48	15.19	30	16.5%	0.27 [-0.24, 0.79]
Klement 2021(b)	64.69	14.27	18	62.79	17.89	23	14.3%	0.11 [-0.50, 0.73]
Ok 2018	38.4	8.8	10	42.4	6.9	9	9.5%	-0.48 [-1.40, 0.44]
**Total (95% CI)**			**145**			**193**	**100.0%**	**0.15 [-0.21, 0.51]**
Heterogeneity: Tau² = 0.14; Chi² = 14.54, df = 6 (P = 0.02); I² = 59%								
Test for overall effect: Z = 0.82 (P = 0.41)								

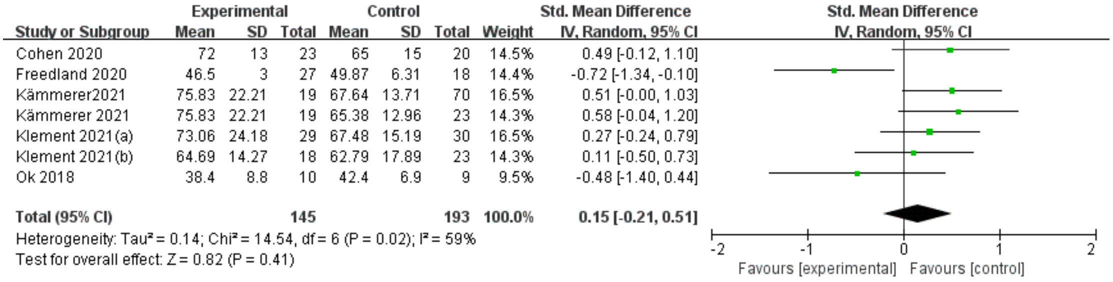

Supplementary Figure 16. Triglycericle (TG)

Study or Subgroup	Experimental Mean	Experimental SD	Experimental Total	Control Mean	Control SD	Control Total	Weight	Std. Mean Difference IV, Random, 95% CI
Cohen 2020	97	33	23	111	46	20	17.4%	-0.35 [-0.95, 0.26]
Freedland 2020	125.61	17.78	27	95.64	13.45	18	16.8%	1.82 [1.10, 2.53]
Klement 2021(a)	80.24	24.43	29	108.53	55.63	30	17.8%	-0.65 [-1.17, -0.12]
Klement 2021(b)	81.32	58.19	18	136.9	36.55	23	17.1%	-1.15 [-1.82, -0.48]
Ok 2018	125.7	49.8	10	110.5	51.1	9	15.7%	0.29 [-0.62, 1.19]
Santos 2018	154.4	93.3	9	99.1	42	8	15.2%	0.71 [-0.28, 1.70]
**Total (95% CI)**			**116**			**108**	**100.0%**	**0.09 [-0.78, 0.95]**
Heterogeneity: Tau² = 1.02; Chi² = 45.25, df = 5 (P < 0.00001); I² = 89%								
Test for overall effect: Z = 0.20 (P = 0.84)								

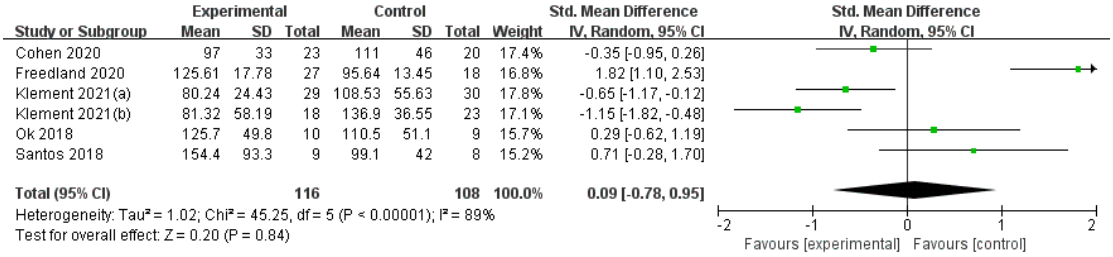

Supplementary Figure 17. C-reactive protein (CRP)

Study or Subgroup	Experimental Mean	Experimental SD	Experimental Total	Control Mean	Control SD	Control Total	Weight	Std. Mean Difference IV, Random, 95% CI
Budipramana 2020	3.82	0.46	12	2.96	0.46	12	7.2%	1.81 [0.83, 2.78]
Cohen 2018	2	1.44	23	3	1.34	20	10.9%	-0.70 [-1.32, -0.08]
Freedland 2020	2.18	0.58	27	2.41	0.78	18	11.1%	-0.34 [-0.94, 0.26]
Kämmerer2021	2.05	2.74	19	1.6	2.17	70	12.3%	0.19 [-0.31, 0.70]
Kämmerer 2021	2.05	2.74	19	1.96	1.94	23	11.0%	0.04 [-0.57, 0.65]
Khodabakhshi 2021	12	13	30	14.3	14	30	12.3%	-0.17 [-0.68, 0.34]
Khodabakhshi 2021(b)	11	13	30	18.6	18	30	12.2%	-0.48 [-0.99, 0.04]
Klement 2021(a)	3.98	5.95	29	3.34	5.27	30	12.2%	0.11 [-0.40, 0.62]
Klement 2021(b)	4.33	2.74	18	12.22	18.41	23	10.8%	-0.55 [-1.18, 0.07]
Ok 2018	25.6	36.6	10	10	11.8	9		Not estimable
**Total (95% CI)**			**207**			**256**	**100.0%**	**-0.08 [-0.43, 0.27]**

Heterogeneity: Tau² = 0.19; Chi² = 25.12, df = 8 (P = 0.001); I² = 68%
Test for overall effect: Z = 0.46 (P = 0.64)

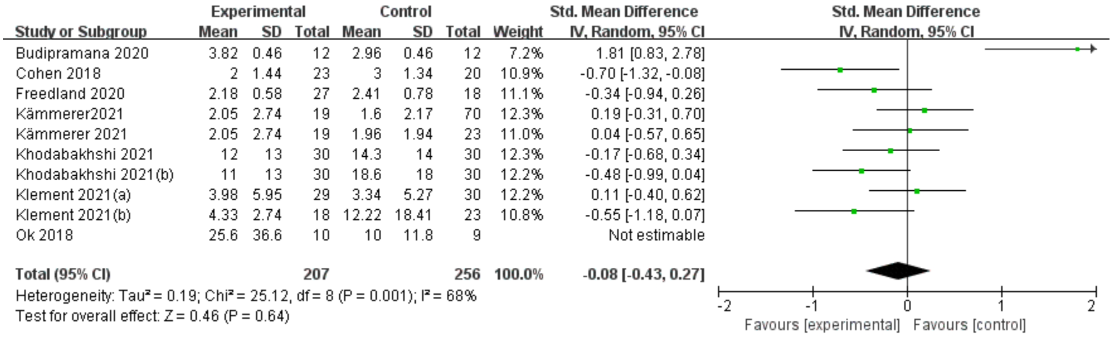

Supplementary Figure 18. IGF-1

Study or Subgroup	Experimental Mean	Experimental SD	Experimental Total	Control Mean	Control SD	Control Total	Weight	Std. Mean Difference IV, Random, 95% CI
Cohen 2018	100.7	44	23	111	59.9	20	12.2%	-0.19 [-0.80, 0.41]
Kämmerer2021	13.99	12.27	19	6.94	3.54	70	13.0%	1.09 [0.56, 1.62]
Kämmerer 2021	13.99	12.27	19	7.83	5.08	23	11.9%	0.67 [0.04, 1.29]
Khodabakhshi 2020(b)	140	63	30	136	34	30	13.3%	0.08 [-0.43, 0.58]
Khodabakhshi 2021	133	61	30	150	48	30	13.2%	-0.31 [-0.81, 0.20]
Klement 2021(a)	187.24	92.53	29	186.46	63.72	30	13.2%	0.01 [-0.50, 0.52]
Klement 2021(b)	135.77	47.21	18	175.72	79.33	23	11.8%	-0.58 [-1.21, 0.05]
Voss 2020	179.9	79.7	22	219.6	104.3	15	11.4%	-0.43 [-1.09, 0.23]
**Total (95% CI)**			**190**			**241**	**100.0%**	**0.05 [-0.34, 0.44]**

Heterogeneity: Tau² = 0.23; Chi² = 26.77, df = 7 (P = 0.0004); I² = 74%
Test for overall effect: Z = 0.25 (P = 0.80)

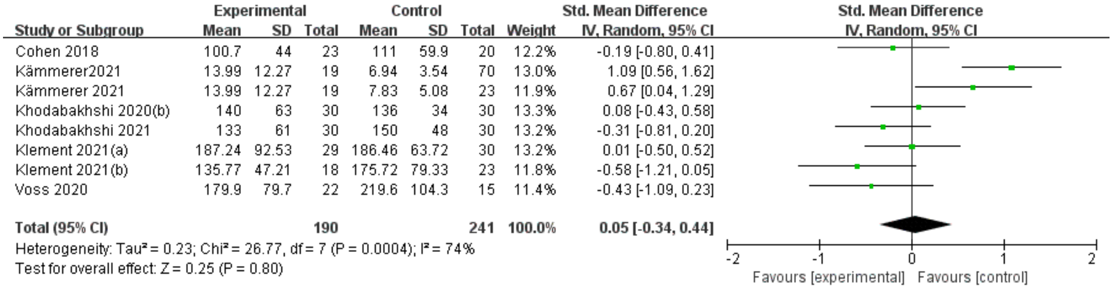

Supplementary Figure 19. TNF-α

Study or Subgroup	Experimental Mean	Experimental SD	Experimental Total	Control Mean	Control SD	Control Total	Weight	Std. Mean Difference IV, Fixed, 95% CI
Kang 2019	498.43	813.3	9	666.52	829.14	9	13.1%	-0.19 [-1.12, 0.73]
Khodabakhshi 2020(b)	19	9.1	30	16.4	6	30	43.2%	0.33 [-0.18, 0.84]
Khodabakhshi 2021(a)	18	8.6	30	17.3	7.3	30	43.8%	0.09 [-0.42, 0.59]
**Total (95% CI)**			**69**			**69**	**100.0%**	**0.16 [-0.18, 0.49]**

Heterogeneity: Chi² = 1.09, df = 2 (P = 0.58); I² = 0%
Test for overall effect: Z = 0.91 (P = 0.36)

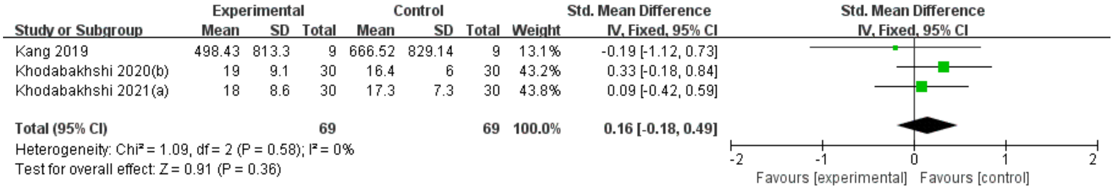

Supplementary Figure 20. Creatinine

Study or Subgroup	Experimental Mean	Experimental SD	Experimental Total	Control Mean	Control SD	Control Total	Weight	Std. Mean Difference IV, Random, 95% CI
Budipramana 2020	0.87	0.19	12	0.72	0.21	12	17.5%	0.72 [-0.11, 1.55]
Kang 2019	232	93.5	9	150.2	49.3	9	14.9%	1.04 [0.04, 2.04]
Khodabakhshi 2021(a)	5.7	4	30	6.9	4.5	30	23.3%	-0.28 [-0.79, 0.23]
Klement 2021(a)	0.76	0.11	29	0.84	0.12	30	23.0%	-0.69 [-1.21, -0.16]
Klement 2021(b)	0.87	0.17	18	0.91	0.19	23	21.3%	-0.22 [-0.83, 0.40]
**Total (95% CI)**			**98**			**104**	**100.0%**	**0.01 [-0.54, 0.56]**

Heterogeneity: Tau² = 0.27; Chi² = 13.95, df = 4 (P = 0.007); I² = 71%
Test for overall effect: Z = 0.05 (P = 0.96)

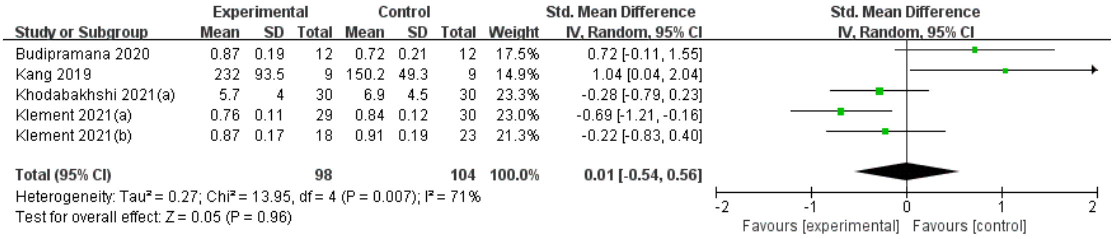

Supplementary Figure 21. Urea

Study or Subgroup	Experimental Mean	Experimental SD	Experimental Total	Control Mean	Control SD	Control Total	Weight	Std. Mean Difference IV, Fixed, 95% CI
Klement 2021(a)	4.74	0.1	29	4.74	0.99	30	42.4%	0.00 [-0.51, 0.51]
Klement 2021(b)	5.14	0.88	18	5.93	1.93	23	28.1%	-0.50 [-1.12, 0.13]
Voss 2020	30.67	8.4	20	30.36	9.67	21	29.5%	0.03 [-0.58, 0.65]
**Total (95% CI)**			**67**			**74**	**100.0%**	**-0.13 [-0.46, 0.20]**

Heterogeneity: Chi² = 1.83, df = 2 (P = 0.40); I² = 0%
Test for overall effect: Z = 0.76 (P = 0.44)

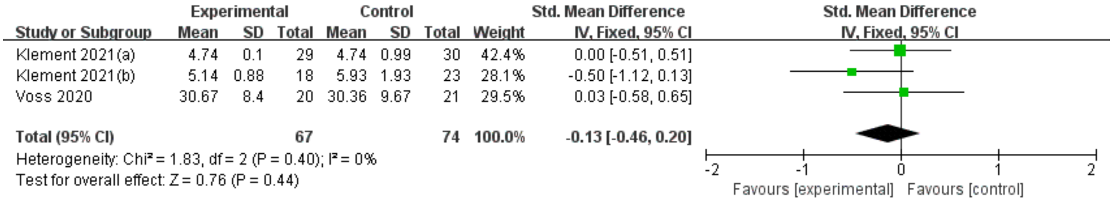

Supplementary Figure 22. Energy uptake

Study or Subgroup	Experimental Mean	Experimental SD	Experimental Total	Control Mean	Control SD	Control Total	Weight	Std. Mean Difference IV, Random, 95% CI
Cohen 2020	1,239	304	23	1,533	304	15	20.2%	-0.95 [-1.64, -0.26]
Kämmerer2021	32.5	1.5	19	24.3	0.7	70	19.7%	8.80 [7.38, 10.21]
Kämmerer 2021	32.5	1.5	19	26.4	1.2	23	19.9%	4.46 [3.28, 5.63]
Khodabakhshi 2021	1,254.32	141.89	30	1,603.8	65.68	30	20.2%	-3.12 [-3.89, -2.35]
Ok 2018	61.31	19	10	38.5	21.9	9	20.0%	1.07 [0.09, 2.05]
**Total (95% CI)**			**101**			**147**	**100.0%**	**2.01 [-1.48, 5.51]**

Heterogeneity: Tau² = 15.61; Chi² = 277.83, df = 4 (P < 0.00001); I² = 99%
Test for overall effect: Z = 1.13 (P = 0.26)

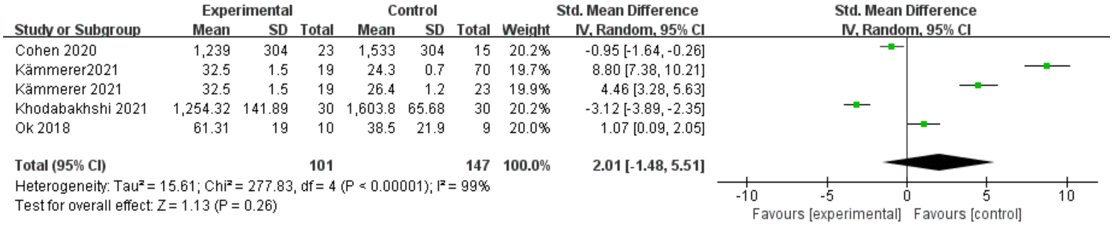

Supplementary Figure 23. Dietary intervention age

Study or Subgroup	Experimental Mean	Experimental SD	Experimental Total	Control Mean	Control SD	Control Total	Weight	Std. Mean Difference IV, Fixed, 95% CI
**6.1.1 > 60**								
Cohen 2018	61.5	8.5	25	58.6	11.7	20	21.1%	0.28 [-0.31, 0.87]
**Subtotal (95% CI)**			**25**			**20**	**21.1%**	**0.28 [-0.31, 0.87]**
Heterogeneity: Not applicable								
Test for overall effect: Z = 0.94 (P = 0.35)								
**6.1.2 <60**								
Kämmerer 2021	52.5	6.4	29	51.3	8.1	92	42.2%	0.15 [-0.26, 0.57]
Khodabakhshi 2021	44.8	8.4	30	45.2	15	30	28.8%	-0.03 [-0.54, 0.47]
Ok 2018	57.8	7.3	10	66.3	9.8	9	8.0%	-0.95 [-1.91, 0.01]
**Subtotal (95% CI)**			**69**			**131**	**78.9%**	**-0.02 [-0.33, 0.28]**
Heterogeneity: Chi² = 4.24, df = 2 (P = 0.12); I² = 53%								
Test for overall effect: Z = 0.16 (P = 0.87)								
**Total (95% CI)**			**94**			**151**	**100.0%**	**0.04 [-0.23, 0.31]**

Heterogeneity: Chi² = 5.07, df = 3 (P = 0.17); I² = 41%
Test for overall effect: Z = 0.29 (P = 0.77)
Test for subgroup differences: Chi² = 0.83, df = 1 (P = 0.36), I² = 0%

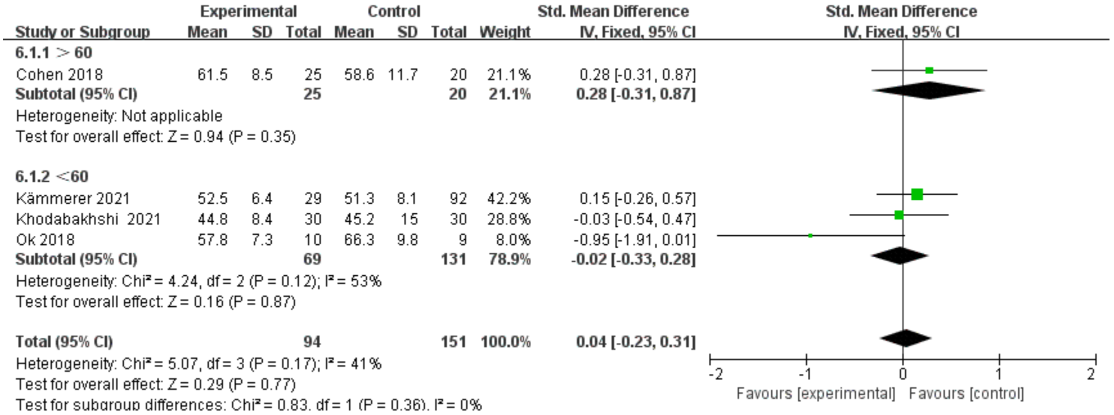

Supplementary Figure 24. Funnel plot

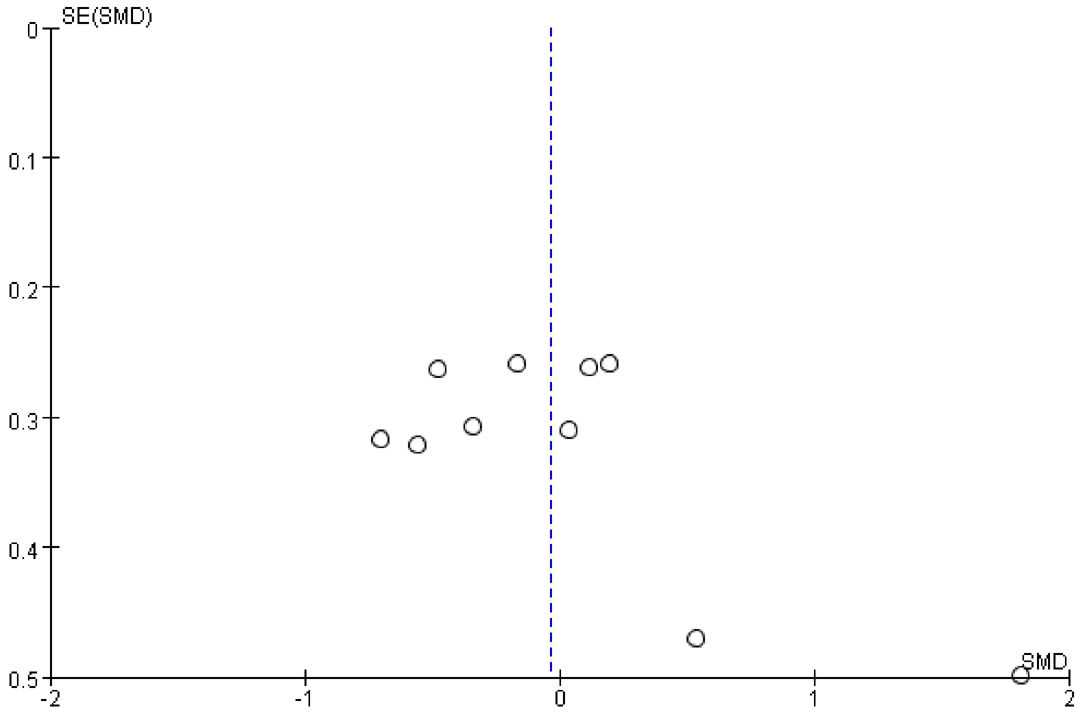

Supplement: Supplementary file 1 [file Image_1.pdf]
